# The method behind the unprecedented production of indicators of the presence of languages in the Internet

**DOI:** 10.3389/frma.2023.1149347

**Published:** 2023-05-18

**Authors:** Daniel Pimienta, Álvaro Blanco, Gilvan Müller de Oliveira

**Affiliations:** ^1^Observatory of Linguistic and Cultural Diversity on the Internet, Nice, France; ^2^UNESCO Chair on Language Policies for Multilingualism, Federal University of Santa Catarina (UFSC), Florianopolis, Brazil

**Keywords:** languages, web, Internet, indicators and metrics, methodology, bias, multilingualism, webometrics

## Abstract

Reliable and updated indicators of the presence of languages in the Internet are required to drive efficiently policies for languages, to forecast e-commerce market or to support further researches on the field of digital support of languages. This article presents a complete description of the methodological elements involved in the production of an unprecedented set of indicators of the presence in the Internet of the 329 languages with more than 1 million L1 speakers. A special emphasis is given to the treatment of the comprehensive set of biases involved in the process, either from the method or the various sources used in the modeling process. The biases related to other sources providing similar data are also discussed, and in particular, it is shown how the lack of consideration of the high level of multilingualism of the Web leads to a huge overestimation of the presence of English. The detailed list of sources is presented in the various annexes. For the first time in the history of the Internet, the production of indicators about virtual presence of a large set of languages could allow progress in the fields of economy of languages, cyber-geography of languages and language policies for multilingualism.

## 1. Introduction

The measurement of the space of representation of languages on the Internet is yet to fascinate the crowds, however, the stakes in terms of the linguistic, cultural, socio-economic and geopolitical levels, are far from neutral.

Concerning the situation of languages in the world, among the estimated 7,000 still existing languages some 40% are endangered^1^ and the intensity of their presence on the Internet could be a meaningful predictive indicator. In order to define efficient public policies for languages, measuring the current situation and its evolution is a prerequisite, in particular as regards to the capacity to assess the impact of those policies.

In the early stages of the Internet, some researchers addressed a new field called *cyber-geography*, which is the study of the spatial nature of computer communications networks.^2^ The acquisition of indicators of the presence of a wider number of languages in the Internet allows us to propose the concept of *cyber-geography of languages* as a related notion (Pimienta, 2021; Pimienta and Oliveira, 2022a,b).

Despite the Internet not being a homogeneous territory from the point of view of its functioning and governance (O'Hara and Hall, 2018), we can treat it as a *multilingual reticular cyberterritory*, analyzing the distribution and interaction between languages in a general space. From a second perspective, however, each language is a territory which guides the densification of relations, including political and economic ones. Each linguistic territory is at the same time a market, with specific production and consumption capacities.

This territorial vision allows us to include in the discussion another relevant concept: the *geopolitics of languages and multilingualism*. Geopolitics is constituted mainly by three factors: *the territory*, which implies location; *the population*, which in this case are the connected speakers of each language; and *the leverage*, which in this case is the digital equipment of each language, its mass of contents and its promotion policies, that is, its ability to receive investments (Flint, 2021).

From this perspective, linguistic cyberterritories are markets in political and economic dispute (Bauböck, 2015).

Several economists have analyzed the economic value of languages from different perspectives (Grin and Vaillancourt, 1997; Gazzola, 2015). But despite the available instruments showing that languages are fundamental for all categories of the service economy described by the WTO,^3^ responsible for an increasing part of the GDP of countries in advanced capitalism, governments and investors have been slow to develop more contemporary perspectives on language management.

In 2020, e-commerce alone accounted for 20% of total global retail sales,^4^ and platforms must communicate in the language of their customers to maintain their market competitiveness (see various sources in Annex 8 of Supplementary material). Whoever manages to penetrate the different language markets will increase their profits, which leads top companies to invest in multilingual strategies (Oliveira, 2010). A language “commodification” process is underway and data on the presence of languages on the Internet is essential for decision-making in this field (Heller, 2010).

Since 2011, policy makers and linguistic researchers had to rely exclusively on two available sources, both originating from the domain of business marketing area, for evaluating the impact of their policies or sustaining their theories.

✓ W3Techs offers the percentage of Internet contents per language,^5^ for the 40 top languages, with a daily update, and also maintains the percentage history.^6^
✓ InternetWorldStats reports the percentages of connected speakers to the Internet for the 10 first languages^7^, with a yearly update.

The analysis of W3Techs' method reveals severe biases that result from not considering the important amount of multilingualism prevailing in the Web (see 4.2 W3Techs biases). The computations of InternetWorldStats rely on the combination of percentage of connected people per country, a trustable figure that is released yearly by the International Telecommunications Unit^8^ (ITU), the United Nations body that reports telecommunications statistics, and demo-linguistic data for L1 (first language) and L2 (second language) speakers per country. The existing sources on demo-linguistic data report large differences, especially in terms of the L2 figures; among them, Ethnologue is generally considered the most reliable source; however, this source is proprietary and not free of charge.^9^

Since March 2022, the Observatory of Linguistic and Cultural Diversity in the Internet^10^ (the Observatory hereafter) offers both these indicators, and meaningful additional indicators, for the 329 languages encompassing a population of L1 speakers exceeding one million (see results in Pimienta, 2022), with plans for yearly updates.^11^ This is the outcome of a long process of bias depuration of a method defined in 2017,^12^ which finally yields outputs with an acceptable threshold of reliability.

The Observatory is not a newcomer in this field: it has been conducting a series of pioneering measurements of Web contents in the English, German, and Latin Languages (French, Italian, Portuguese, Spanish, and Romanian), between 1997 and 2007 (Pimienta et al., 2009). The method leveraged the total of word or expressions occurrences in Webpages, which was reported by search engines exploring a large percentage of the Webspace. The Observatory was obliged to resign, after 2007, when search engines stopped reporting trustable figures and the proportion of indexed webpages was considerably reduced.

The new method, developed in 2017, which allowed for the design of a set of indicators for the 139 languages with more than 5 million L1 speakers, ushered in a new approach, defined in 2012 and applied for single languages, mainly French (Pimienta, 2014) and Spanish (Pimienta and Prado, 2016). This approach focused on the management of a set, as large as possible, of disperse sources of figures about languages or countries with a certain type of relationship with the Internet. This relation could be direct (e.g. repartition per country of subscribers to a specific social network or languages supported in on-line translation services) or indirect (e.g. ranking in the e-commerce domain or the average number of mobile per person in each country). With the notable exception of the Wikimedia Foundation offering figures for each of the provided services and for the 327 supported languages,^13^ the scarcity of figures related to languages used in the Internet was compensated by using figures related to countries, which were more numerous, and these were transformed into figures per language by weighting with the demo-linguistic data. The collected figures were organized into different categories: *contents, traffic, usages, indexes*,^14^ and *interfaces*.^15^ In 2017, with mathematical coherence and using statistical techniques to extrapolate missing data, the method was generalized for many languages, beyond French or Spanish. A model was designed to process the whole set of sources into meaningful indicators for the 139 languages with L1 speakers numbering over 5 million.

Thereafter and since 2017, the work was essentially dedicated to the struggle against the various **biases** proper of the method or the data sources. In 2021, this resulted in a Version 2 (Pimienta, 2021) with the same structure but with certain important biases controlled, in particular, through the usage of the Ethnologue Global dataset 24 (March 2021) for demo-linguistic data. Subsequently, the language coverage was extended to the 329 languages with L1 speakers numbering over one million. The pursuit of the fight against biases continued and, in March 2022, led to a final redefinition of the approach and the confidence that a reasonable level of control of biases had been attained, with the capacity to produce reliable figures, within a confidence interval of -+20%, an empirical estimation not sustained through any statistic computation.

Why is it so important to identify the biases and, as far as possible, try to mitigate them or, if not possible, evaluate the impact on the results obtained from those biases which cannot be overcome? In any research activity the scientific method calls for careful use of data and statistics as biases may occur, and, if they are heavy, can totally discredit the obtained results. While this is a known evidence in health matters, where a large quantity of statistical studies is conducted, either to evaluate the effect of some treatment or to measure the prevalence of a particular disease in a specific population, that the sampling shall be carefully selected and the method should rely on solid grounds (for instance with double blind procedures, in which neither the participants nor the researcher knows which treatment or intervention participants are receiving), this concern about biases must apply equally to all field of research.

The field of measuring languages in the Internet is at the intersection of two areas where biases are quite prevalent: demo-linguistics (linguistic demography) and the Web. In both areas there is no strong consensus on the data and large differences could occur, depending on sources, on figures such as how many speakers of that language reside in that country or what is the total number of webpages.

Biases may happen in different manners, proper of the sources of data used, inherent of the method used, in the selection made for a sampling, in computational hypothesis or in the hypothesis sustaining some necessary simplifications. While it is the main responsibility of the producer of data to take systematic care of the possible biases and document those remaining, it is also the responsibility of the researcher using those data to identify the sources and check their credibility, find the description of the method and analysis its possible bias, all that prior to drawing conclusions based on those data. Good reasoning on wrong data will hardly produce reliable conclusions! The ease provided nowadays by search engines to identify public sources for specific data in the Web does not cancel the need for checking those sources, still more today with an evolution of the ranking of search engines results giving more tribute to marketing considerations than to scientific rigor…

The theoretical standard method to measure the space of languages in the Web is to crawl all the webpages of the Internet and to apply to each of them an algorithm of language recognition and count each page language(s), paying attention that a single page could hold more than one language. Finally, dividing the count of each language by the total number of crawled pages give the percentage. Prior to that process, the possible biases of the language recognition algorithm need obviously to be analyzed.

According to Netcraft,^16^ there are today over 1.2 billion websites of which 200 million are active. One source^17^ evaluates the total number of webpages around 50 billion, of which less than 10% would be indexed by search engines. In that context, targeting websites instead of webpages is a simplification used by most of the studies, which implies some new risks of biases to be considered, still more if the language recognition is applied exclusively to the home page of each site, which quite often have English components even for non-English websites. Yet the universe of the complete list of existing websites is a crawling option that not even the search engines are in capacity to handle; other simplification is then required, practically to select a reduced sampling which would be hopefully representative of the whole Web. This is another risk of bias which a rapid view at the history of trials will highlight.

Before 2007, the number of initiatives to try to measure the percentage of presence of languages in the Web have been limited; hereafter is a fast exploration of them, a deeper analysis can be read in Pimienta et al. (2009).

From the three first attempts, in the period 1995–1999, one used the standard approach (Babel team, a joint initiative from Alis Technologies and the Internet Society^18^) and the two others (Grefenstette and Noche, 2000) and the Observatory^19^ used different approaches. Grefenstette and Noche (2000) used a technique for estimating the size of a language-specific corpus from the frequency of commonly occurring words in that corpus and applied it to the Web. The Observatory compared the number of occurrences of an equivalent vocabulary in the different languages studied (data provided by search engines). The Babel team defined its Web sampling to be analyzed by a technic of randomization of IP numbers which finally consisted in little more than 3000 websites on the home page of which the language recognition was applied. There were many causes for biases but the major issue is that only one sampling, and therefore one unique measurement, was realized. In statistical terms the absence of a series of measurements invalidates the results because a unique sampling of 3000 websites upon a universe, at that time, of one million, is totally irrelevant. The valid approach should have been to replicate the operation, say hundred times at least, and compute average, variance and other statistical attributes of the obtained distribution. The fact is however that this flaw approach was reused two times (Lavoie and O'Neill, 1999 and O'Neill et al., 2003), and conveyed to medias the erroneous idea that 80% of Web contents were in English, without change during the period 1996–2003.

In the same period, the Observatory improved its method with the collaboration of linguists from a partner institution, based on equivalent vocabularies in different languages, focusing and avoiding as far as possible the potential biases. The Observatory presented results showing English declining steadily from 80% of the Web contents, in 1996, to 50%, in 2007. This approach, although limited to Latin languages, English and German, produced a consistent series of measurements in the period; however its dependency on the reliability of search engines world occurrence counting triggered its end in 2007.

Two other initiatives occurred in the period, both using the standard approach: The Language Observatory Project - LOP (Mikami et al., 2005), and a project from the Statistics Institute of Cataluña - IDESCAT (Monrás et al., 2006). The LOP project, an academic consortium with partners joining forces in the two main requirements, Web crawling with strong capacity and modern language recognition algorithm, presented all the attributes to become the best solution to address the theme, with the rigor of academic researchers and the strength of crawling capacity. It started focusing languages in the less populated Asian countries and expanded progressively. A collaboration was set with the Observatory, under the umbrella of the World Network for Linguistic Diversity - MAAYA,^20^ when LOP produced data for Latin American countries, but unfortunately, this project, which was coordinated by Nagaoka University, came to an end shortly after the earthquake and tsunami which affected Japan in 2011. As for the IDESCAT project, which focused Catalan language specifically, it had a short life duration. This period of academic activities around the theme has been followed by a practical leave of that field to marketing companies, with, as consequence, the reign of non-fully transparent and non-peer reviewed methodologies and, at the same time, excellent marketing allowing large public impact.

After 2007, apart from the Observatory initiatives, a consortium of Greek universities (Giannakoulopoulos et al., 2020) used the standard approach to evaluate the presence of English in websites under European Union countries code top level domains (ccTLD). Their crawled sampling includes a little more than 100 000 websites and their method paid due attention to multilingualism of websites by systematically checking the language of all internal links from the home page. From their output data it is possible to compute a figure of 28% of English versions of websites for all Union European ccTLD websites (including United Kingdom, Ireland and Malta) or 13% for Non-English-speaking European countries (Pimienta, 2023).

W3Techs applied, until May 2022, its language recognition algorithm, on a daily basis, upon a list of the 20 million most visited websites, provided by Alexa.com, a commercial service of Web traffic analysis. After May 2022, when the Alexa service was stopped, it was applied upon the million most visited websites list provided by Tranco,^21^ a research oriented non-for-profit service self-presented as “*hardened against manipulation*”.

W3Techs applies the algorithm on the home page of each one of the websites of the list and count a single language for each of them, ignoring the potential multilingualism of the websites. The absence of alternative for a long period, and also the deserved reputation of the company for its main service, surveys on Web Technologies, has transformed that source into an extremely popular one and quite often a reference even for the research community. At difference with the other 26 Web Technologies surveyed by the company, such as JavaScript, Markup Languages or data centers, languages are a particular “Web technology” with the property that more than one such “technology” can be associated with a single webpage or a single website. Multilingualism is a property of the Web which requires due consideration in order to provide unbiased results. This property is at the heart of the method exposed hereafter.

## 2. Methods

### 2.1. Overview

The method is an **indirect approximation** of Web contents per language, supported by consistent experimental observations, made since the beginning of the Observatory, which indicate that the ratio between *world percentage of contents* and *world percentage of connected speakers* (ratio defined as *content productivity*) has rarely been measured lower than 0.5 or higher than 1.5 for languages with full digital existence.

This observation suggests the existence of a natural economic law, which links, for each language, the **offer** (Web contents and applications in the given language) to the **demand** (speakers of that language connected to the Internet). When the number of connected persons increases, the number of webpages naturally increases accordingly, in **more or less** the same proportion. This trend occurs because governments, businesses, educational institutions, and certain individuals generate contents and applications to respond to that demand.

Notably, supporting the previous statement, surveys and studies on Internet user's behavior consistently report that Internet users prefer to use their mother tongue when contents are available, especially for e-commerce, and in complement are eager to communicate in their second language(s) (see, in Annex 8 of Supplementary material, a selection of sources to support that claim).

Thus, depending of each language context, there is a type of modulation of the mentioned ratio, which render it, **more or less**, above or below one. Certain languages exhibit a *content productivity* better than those of others, depending on a set of factors proper of the language or related to the different country's context where some proportion of the speakers of that language connects to the Internet. The following factors have been identified.

Factors proper of the language:

- Evidently, the relative amount of L2 speakers, as some people are producers of Web contents in a language different from their mother tongue, for instance for economic reasons.
- The technological support of the language for cyberspace, reflected somehow in its presence in application's interfaces and translation programs, which would make easier or not the content production.

Factors depending on each country with L1 or L2 speakers of this language:

- The amount of Internet traffic, depending on the country's tariff, cultural, or educational context.
- The number of subscriptions to social networks and other Internet popular applications.
- The level of progress of the country in terms of Information Society services (such as e-commerce or government applications for paying taxes).

Therefore, if sufficient and meaningful figures about each of the mentioned factors are collected for creating corresponding indicators, then the value of the *content productivity* ratio can be estimated, and based on the proportion of speakers connected, the *contents* proportion can be deduced.

This forms the core of the method, and it is synthetized in Supplementary Figure 1, which shows the indicators that are processed for each language and the corresponding quantity of sources used by the model.

### 2.2. Description of the inputs of the model

The inputs of the model are split into 5 categories of sources: *internauts, usages, traffic, interfaces*, and *indexes*.

#### 2.2.1. Internauts

This comprises the percentage of L1+L2 speakers connected to the Internet for each language. The transformation of the source' figures, expressed by country, into the required figure, expressed per language, is performed via weighting.

CS(j) represents the percentage of connected speakers for language j.


$$\begin{array}{l} CS\left(j\right)=\overset{i=c}{\underset{i=1}{\sum }}SP\left(i,j\right)xCC\left(i\right)/\overset{i=c}{\underset{i=1}{\sum }}SP\left(i,j\right) \end{array}$$

where

- c indicates the total number of countries.
- SP (i, j) denotes the number of L1+L2 speakers of language j in country i.
- CC (i) represents the percentage of connected persons for country i.

The matrix product CS = SP + . x CC in APL^22^ notation or = SumProduct (SP;CC) in Excel notation, is a weighting operation having, as input, data per country and, as output, data per language.

The validity of this computation stands on the implicit hypothesis that, within the same country, all language groups share the same figure for the percentage of connected persons. This is a founding bias of the method analyzed in chapter 2.4.1.

The vector CS(j) is a key element of the model which serves, in weighting operations with various sources, to compute the modulation of each indicator.

The source for the SP matrix is Ethnologue; the model uses Ethnologue Global Dataset #24 of March 2021. The sources for the CC matrix are the ITU ^23^ and the World Bank; the ITU, the historical source of these figures, relies on government reports or its own estimation (when the former is not available). As the ITU stopped reporting its own estimations in 2017, the source is completed by using figures^24^ from the World Bank, which fills that gap in many cases. When no recent figures are available, an extrapolation of older figures is used in the model.

As the technique of weighting will be utilized as applicable in the model computations, an issue emerges: the major drawback is that it does not yield credible results if all countries are not filled because the matrix product transforms this absence by zero, which unacceptably penalizes results for languages with strong presence in countries for which the source indicates no information. Therefore, the solution involves extrapolating the missing results prior to computations (see 2.5.2 Extrapolation).

#### 2.2.2. Interfaces

Researchers from the MetaNet network^25^ are adequately analyzing the technological support for European languages; however, certain types of metrics are unavailable for evaluating the technological support for all languages in the world. To approximate this parameter, the focus has been placed on the presence of each languages as an option in the interface of a set of popular Internet applications and as one of the pairs in on-line translation services. Sixteen elements have been identified wherein the list of supported languages is accessible. The list of measured applications is reported in Annex 3 of Supplementary material. It is to be noted that a new metrics seems to have emerged after we have concluded version 3 and is an alternative to consider for next version (Simons et al., 2023).

#### 2.2.3. Indexes

Herein, the theme involves rating countries in regards to their progress on fulfilling the Information Society's criteria. A further weighting with demo-linguistic data will transform this figure into a rating of languages. In version 1, a list of 4 sources was used. Starting with version 2, a systematic search was realized, and 27 sources were identified, thereby rendering the selection almost exhaustive (see Annex 4 of Supplementary material).

#### 2.2.4. Usages

Five sub-indicators have been identified, and corresponding sources have been used:

- Subscribers to social networks: 36 sources have been utilized, each related to social networks with more than 100 million subscribers. For the main occidental social networks, direct figures on subscribers per country have been identified. For the remaining social networks, mostly from Asia, partial traffic figures per country have been leveraged, using Similarweb,^26^ extrapolated to the rest of countries, proportionally to the percentage of connected persons per country.
- E-commerce: a single source has been employed, which completes the job perfectly: the T-index indicator from the Imminent Translated Research Center.^27^ This indicator ranks countries according to their potential for online sales, thereby estimating the market share proportion of each country in relation to global e-commerce. The set of percentage per country is transformed by weighting with the connected speakers per language into a set of percentage per language. Notably, Imminent also yields the set of percentages per language, probably with a similar operation. Moreover, there are slight differences between Imminent and Observatory's computations, presumably resulting from different demo-linguistic data. The model utilizes Observatory's data instead of the direct Imminent source, because Imminent is limited to 89 languages, whereas observatory's extrapolation technique allows for encompassing all the languages of the study.
- Video streaming: the model leverages only two sources at this stage: the percentage of Netflix subscribers per country and the YouTube penetration per country.
- Open contents: the model makes use of only one source at this stage: the percentage per country of the sum of 2012/21 % OpenOffice downloads.
- Infrastructure: the model uses three key World Bank's figures, which are merged into the following two indicators: % fixed broadband subscribers per country and % fixed telephone + mobile subscribers per country.

The final results have been first weighted to reflect the current trust in the figures,^28^ thereby reducing the biases, with the following values:

- Subscribers to social networks: 0.3
- E-commerce: 0.3
- Video streaming: 0.05
- Open contents: 0.05
- Infrastructure: 0.3

The results are then transformed by weighting into repartition per language.

The detailed list of sources for *usages* is in Annex 1 of Supplementary material.

#### 2.2.5. Traffic

Tools (such as Similarweb) are available for obtaining an estimate of traffic repartition per country to any specific website. In general, these tools offer data for websites ranked within the first million or ten million most visited sites. The challenges herein involve evaluating these tools and understanding their potential bias to establish a selection of websites with minimum bias, while maintaining a workable size (says less than or around 1,000). Several changes were implemented from version 1 to version 3 to overcome the biases; these changes are described in section 2.4.5 (Traffic). The complete list of websites used for *traffic* is presented in Annex 5 of Supplementary material.

#### 2.2.6. Contents

Contents was an input of the model for the two first versions, as the original methodological objective was to collect a maximum of sources and Wikimedia, which collected, for each of its applications,^29^ and for each supported language, reliable and interesting statistics per language, notwithstanding the fact that it is probably the more multilingual application of the Web with its 327 linguistic versions. Version 3 decided to cancel this indicator from the input list. The chapter 2.4.8 (Contents) discusses the corresponding biases and reports the rationale for that decision.

### 2.3. Description of the outputs of the model

The model yields the following outputs, for each language:

- *Speakers*: the share of world L1+L2 speakers.
- *Connected Speakers*: the percentage of L1+L2 speakers connected to the Internet.
- *Internauts*: the total share of L1+L2 connected speakers expressed in percentage.
- *Contents*^30^: the total share of Web contents expressed in percentage.
- *Virtual presence*: the ratio of *contents* over *speakers*. The world value (and average) is 1: a value higher than 1 implies a virtual presence that is greater than the real-life presence.
- *Content productivity:* the ratio of *contents* over *internauts*. The world value (and average) is 1: a value greater than 1 implies high productivity of connected speakers.
- *Cyber-globalization index*:

*CGI* (*L*) = (*L*1 +*L*2)/*L*1(*L*) *x S*(*L*) *x C*(*L*) where:

- L1+L2/L1 (L) denotes the ratio of multilingualism of language L (from Ethnologue source).
- S(L) indicates the percentage of world countries with speakers of language L (from Ethnologue source).
- C(L) symbolizes the % of speakers of language L connected to the Internet (computed using the model).

CGI is an indicator of the strategic advantages of a language in cyberspace.^31^

In addition, Table 1 (*Cyber-geography of languages*) is generated by grouping the previous indicators according to language families (using the definition of Ethnologue), thereby yielding an interesting global perspective on the situation and trends.

**Table 1:** Cyber geography of language families.

**Languages from**	**Africa**	**Americas**	**Arab world**	**Asia**	**Europe**	**Pacific**	**Not included**	**Total**
Speakers L1+L2	9.21%	0.31%	3.53%	48.24%	30.91%		7.81%	100%
Internauts %	29.8%	56.7%	64.0%	49.3%	82.6%		47.06%	56.91%
% from internauts	5.21%	0.32%	3.89%	44.63%	39.51%		6.36%	100%
Contents	2.89%	0.22%	3.09%	44.77%	45.39%		3.64%	100%
Virtual presence	0.31	0.71	0.88	0.93	1.47		0.47	1
Contents productivity	0.56	0.69	0.79	1.00	1.15		0.57	1
Number of languages	138	8	1	135	47	0		329

### 2.4. Analysis of biases

Table 2 depicts the evolution of biases from V1 to V3 using a subjective rating from 0 (biases so huge that data is meaningless) to 20 (absolutely free of biases), with a rating of 10 (notable but bearable biases) in the middle.

**Table 2:** Bias assessment.

**BIAS assessment rate over 20**	**V1 2017**	**V2 2021**	**V3 2022**
Core method	17	17	17
Method for L2	13	19	19
Internauts	19	16	19
Indexes	15	18	18
Contents	5	8	OUT
Trafic	13	11	17
Interfaces	19	19	19
Usages	12	12	16

#### 2.4.1. Core of the method

The implicit bias of the core of the model involves considering that all the languages in the same country share the same rate of connectivity to the Internet (the national value reported by the ITU). Observably, the ground reality is different because the concept of digital divide also exists within each country.

This working hypothesis provokes a positive bias for speakers of non-European languages living in developed countries (who are probably less connected than the average), and reciprocally, a negative bias for European languages speakers in developing countries (who are probably more connected than the average). As the foundation of the method, this hypothesis cannot be changed; the decisions taken to deal with it are:

1) comparisons between language's performance within a country are not allowed;
2) as the risk of important bias grows inversely proportionally to the size of the speaker's population, the study was first limited to languages with more than 5 million L1 speakers and later extended to languages with more than 1 million L1 speakers. Future versions may try to extend this threshold but probably never to a value below 100 000 as the effect of this bias could become unavoidable.

#### 2.4.2. Method for L2

For the first time, in 2021, Ethnologue extended its demo-linguistic data per country to L2 speakers. This allowed for the removal of one of the most important biases of the method (in V1), which resulted from the extrapolation of data (for example percentage of connected speakers) from L1 to L2, a process which biases positively the results of languages with high presence in developing countries, such as English and French. Indeed, this process assigned Internet connection rates of L2 speakers in developing countries that are higher than those in reality. Starting in V2, with the existence of demo-linguistic data per country for L2 as well as L1, the core method is directly applied to L1+L2 populations; this extrapolation bias disappears, but obviously not the core method bias, which apply the same for L1, L2 and L1+L2.

In particular, the demo-linguistic source exhibits a larger bias for L2 figures than for L1 figures as there is no perfect definition of the level of mastering of a second language required to be computed as L2. In reality, the L2 figures' sources vary in huge proportion, especially for English: Ethnologue's figure for English is 1.348 billion L1+L2 speakers (L1 = 370 million, L2 = 978 million), whereas other sources propose a value of 1.18 billion^32^ or 1.5 billion.^33^ Moreover, in 2008, David Crystal expressed the possibility of this figure tending to 2 billion.^34^

#### 2.4.3. Internauts

After the demo-linguistic data, this element is the second main element of the model, and it must be rooted in a reliable source. As mentioned in 2.2.1, figures from ITU and World Bank are combined to obtain optimal and mostly reliable up-to-date data.

#### 2.4.4. Indexes

With the extension of the sources in V2 reaching close to exhaustivity and a selection from reliable institutions (international organizations and non-Governmental organizations), the selection bias is minimal, and the trust in the data is maximal.

#### 2.4.5. Traffic

The available tools providing repartition of traffic per country to a large set of websites (the ones considered as the most visited) are: Alexa.com, Similarweb.com, Ahrefs.com, and Semrush.com. All tools are proprietary technology of marketing companies, and these companies are not totally transparent about their method. For instance, Alexa,^35^ the older and most famous tool, performs from a banner that users can download. This banner, associated to a Web browser, reports to Alexa those sites that are visited by the user using this browser. With the collection of all the data sent by all the banners around the world, Alexa designs its outputs, both in terms of ranking sites and traffic repartition per country. Evidently, the geographical repartition of banners could be an indication of probable biases, but unfortunately, this information remains unpublished.

The process of this indicator is the most time-consuming process for overcoming biases. Alexa.com was used in version 1, with a selection of 450 websites. The traffic figure per country from Alexa were compared with the subscriber's figure per country, collected from various sources, revealing that Alexa was positively biased for English and French and strongly biased against Asian countries and Brazil. To combat the unavoidable *selection bias*, the process of the indicator was not realized by simple average but rather by a *reduced mean* with a large value of 20%, thusly attempting to mitigate the selection biases.

Trials in version 2 revealed that Alexa seemed to have corrected the Asian negative bias; however, a new bias appeared that affected the European countries. Further trials lead to the discovery of a bug: the main country in terms of traffic was sometimes not listed, and this could be the reason for the observed bias in the results, as this bug occurs especially for European countries. Subsequently, it was decided that Alexa should only be used when the sum of percentages offered was higher than 70%, a simple strategy for eliminating those mistaken cases. Ahrefs and Semrush were attempted but not used because of a strong bias in favor of English and, for one of these tools, a total of percentages per country often higher than 100%. Similarweb yielded results relatively close to those of Alexa.com, after the mentioned correction, and it was decided that Version 3 would use both tools and retain the figure of half the sum of each.

After several tests and experiments were conducted, it was observed and concluded that the *selection bias* was definitively a serious problem that must be solved through a strategy more drastic than the reduced mean.

Finally, version 3 addressed the situation with a new approach; this approach allowed for the management of a selection of over 1,000 websites wherein the bias was reduced through all possible means. This decision obliged to resign to an interesting but statistically weak outcome of previous versions, which consisted of grouping the websites by theme and drawing tentative conclusions for certain languages about their strength or weakness as related to those themes. The issue about determining if these results reflected the selection biases more than some thematic reality of the language presence in the Internet remained at the same time unsolved.

To achieve the objective of unbiased selection, it was finally decided to list a selection of the most visited websites in each country, with a number of websites proportionate to the country's global traffic. The algorithm was not set to target all the countries for practical reasons but was limited to the 55 countries occupying the top positions in terms of contents for languages spoken in these countries (see Table 3).

**Table 3:** List of countries treated for the selection of national sites.

**Afghanistan**	**Algeria**	**Germany**	**Angola**	**Saudi Arabia**	**Argentina**	**Australia**
Bangladeshi	Belgium	Brazil	Bulgaria	Cambodia	China	Hong Kong
Taiwan	Colombia	South Korea	Egypt	United Arab	Emirates	Spain
United States	France	India	Indonesia	Iran	Iraq	Italy
Japan	Kazakhstan	Kuwait	Lithuania	Malaysia	Mexico	Morocco
Mozambique	Nepal	Nigeria	Uzbekistan	Pakistan	Netherlands	Philippines
Poland	Portugal	Romania	UK	Russia	Singapore	Sudan
Sri Lanka	Tanzania	Thailand	Turkey	Ukraine	Sri Lanka	Vietnam

The rule was set to select, for each country, at least the first three most visited sites including among them, at least, the first local domain (ccTLD,^36^ such as .fr for France). This rule was set to prevent the selection from being heavily concentrated in the most visited sites globally (generally.com). Obviously, this rule could not prevent the most visited global websites (such as Google.com or Facebook.com) from appearing in many country's selection, and a weighting was performed to respect those figures.

To realize the selection, four tools were used (Ahrefs, Alexa, Semrush, and Similarweb); however, on certain occasions, owing to a lack of data for countries with a small population, we had to collect the data from other sources.

Finally, a total of 1,421 websites were selected automatically (the selection process was aided via computer programming to prevent mistakes or unwanted biases), of which 733 were different websites. The number of occurrences of each website in the 1,421 selection was retained for further weighting. For each country, the number of websites corresponding to the proportion of the global Internet-traffic share was also computed and recorded for further weighting, as a strategy for controlling the selection bias.

This method insured the most unbiased possible selection for the *traffic* measurement and overcame the considerable bias against Asian countries, which has existed since the beginning of the study. This method definitively enhanced the final results for Chinese, Hindi, Arabic, and the other Asian languages.

A remaining bias may still exist, which penalizes those countries (and the associated languages) where the general level of digital literacy is the highest, and therefore, there is significant traffic to sites with scientific or literary content, and in any case excluding social networks and other world-famous sites. Unfortunately, this is the trade-off for obtaining results that are free of major biases. It is clear that this marginal bias will not favor the languages of developed countries, which are most often European languages.

As a possible enhancement for version 4, a new indicator could be incorporated by establishing, for each country, the proportion of sites in the national domain compared to sites in the generic domain. This indicator could be a first step toward measuring the global degree of digital literacy by country and could even be used through a new weighting to compensate for the residual bias in question. Nevertheless, the version 3 raw results of the model could slightly disadvantage French and English, and on the contrary, now seem to slightly favor Chinese.

#### 2.4.6. Interfaces

For each language, a ranking is established as the number of times the presence of that language exists in the list of selected applications (interface or on-line translation). Based on this ranking, the weighting operation, with the percentage of connected speakers, generates the “modulated” percentage expected. Obviously, this indicator is remarkably “aggressive” as hundreds of languages are completely absent from the list; thus, these languages are attributed a figure of 0%, implying the complete absence of any technological support. This harsh measurement reflects the crude reality of this field: despite the growing efforts of language technology researchers,^37^ several languages experience a level of digital technological support that is almost non-existent.

#### 2.4.7. Usages

The **subscriber's** element resulted in a strong pro-occidental bias in versions 1 and 2, owing to the absence of non-occidental social networks, and a particular effort has been made in version 3 to complement the 11 initial sources^38^ with analogous applications from the rest of the world.

The criteria, chosen for complementing the sources, involved retaining social networks with more than 100 million subscribers. The data measured is the repartition of subscribers per country; when no source is identified, the data is established from the traffic per country, data obtained from the Similarweb service, and extended to all countries through extrapolation (see 2.5.2 Extrapolation).

The repartition of subscribers per country, after extrapolation of each element, is weighted in function of the total number of subscribers per application and is finally transformed in percentage per language by weighting with the demo-linguistic matrix.

In version 3, the complementation considerably reduced the bias against non-occidental countries and indirectly against non-European languages. The complete list of social networks processed is reported in Annex 1 of Supplementary material.

For the **e-Commerce** element, as mentioned previously, the source is unique but remarkably trustable.

For **video streaming**, the model utilizes only two sources at this stage: the percentage of Netflix subscribers per country and YouTube penetration per country. Clearly, this sub-indicator should be extended in the next release of the model with alternative streaming applications beyond YouTube and Netflix, with a special effort for non-occidental countries. Nevertheless, the element receives a low weighting.

For **open contents**, this sub-indicator clearly needs to be extended in the next release of the model with more data related to openness, especially in the field of MOOCs. Nevertheless, the element receives a low weighting.

For **infrastructure**, the World Bank figures concerning fixed lines, mobile, and broadband per country are quite reliable and offer a sound basis for the indicator. By summing fixed lines and mobile in a single figure, a balance is achieved between developed countries with large fixed-line penetration and developing countries with high mobile penetration.

This indicator needs to be enhanced for the next release. Nevertheless, the main objective of overcoming the occidental bias resulting from working only with the main occidental social networks has been achieved for the social networks component and has produced the expected effects on the results, revealing the booming presence of contents from Asian countries and corresponding languages.

#### 2.4.8. Contents

Along with *usages*, this indicator has received a higher attention in the work against biases. Moreover, this indicator's biases, inherited from the Wikimedia galaxy, had a major influence on the two first versions results, yielding a notable advantage, for indicators independent from speaker's population, to results of the languages with major presence in Wikimedia.

There are two main challenges associated with Wikimedia. First, despite its notable efforts and success in becoming truly global, it does suffer from an occidental bias. Second, some particular languages (like Cebuano, Malagasy or Tagalog) have invested a lot of efforts to participate to the online encyclopedia and show presences that are considerably disproportionate with the reality of their percentage of connected speakers. Other languages, such as Hebrew, Swedish, and Serbo-Croatian, have seen their results in first versions boosted by their heavy presence in Wikimedia services. Furthermore, certain languages have artificially boosted their number of articles by translating these articles from other linguistic versions while maintaining an extremely low rate of updates.

The focus on unbiasing has been set in these directions. In version 2, the following formula was set and used as an indicator, instead of the number of Wikipedia articles, to efficiently remove the mentioned artificial advantage: *W* (*i*) = *Articles* (*i*)*x Edits* (*i*)*x Editors* (*i*)*x Depth*(*i*)/(*L*1+*L*2 (*i*))^2^, where:

- i depicts one of the languages.
- Articles (i) represents the number of Wikipedia articles for language i.
- Edits (i) denotes the number of editions of the articles for language i.
- Editors(i) depicts the number of editors for the articles for language i.
- Depth (i) symbolizes an indicator of the frequency of updates of articles for language i.^39^
- L1+L2(i) indicates the number of first and second language speakers of i.

All elements of the formula are reported in Wikipedia statistics. For more details, see the description of version 2 method.^40^

For version 3, a profound and systematic effort was dedicated to balance the Wikipedia figures with equivalent figures from other languages. The table presented in Annex 2 of Supplementary material lists the online encyclopedias processed with the figure gathered, mainly in terms of the number of articles. Based on this table, the content indicator was designed with a fairer representation of languages by cumulating, by languages, the different number of articles in all online encyclopedias. The conclusion of this heavy, necessary, but finally frustrating effort, was that certain languages (such as Chinese or Turkish) have invested massively in online encyclopedias, whereas other languages do not appear to be interested in this regard. The impact of such drastically different figures on the end indicators produced is extremely high, and finally, evidence emerged that online encyclopedias are not honest witnesses of the reality of Web contents and should not be used in the model.

It was a real dilemma to abandon these wonderful statistics of Wikimedia; however, the suppression of *contents* as an input data resulted in the positive renewal of the conception of the approach into a bias-light and coherent model.

Instead of naming *power* the main output of the model (computed as the average of all indicators), it was renamed directly as **contents**. The output indicators named *capacity*, and *gradient* in version 1 and 2 were conserved with the same arithmetic operation and renamed **virtual presence indicator** and **content productivity indicator**, which become more natural and understandable concepts. Moreover, all the weighting operations developed inside the model from version 1 were now reflected coherently in the conceptualization of the approach, as a modulation of content productivity. Concurrently, the aforementioned anomalies of the results, which were driven by the particularities of Wikimedia, disappeared, leaving room for more trustable and predictable results. A notable symptom is that Japanese surged to the first place in terms of the *virtual presence* and *content productivity*, which is coherent with the pervasive real-time use of the Internet in Japan. Some of the languages favored in previous versions by their high presence in Wikipedia remain in high positions in version 3, but not at the first positions; this keeps validate the statement that languages of countries (or regions) with highest performance in Information Society parameters benefit from good positions in terms of the *virtual presence* or *content productivity* indicators.

### 2.5. Model

#### 2.5.1. Pre-processing

The main part of the data reported by Ethnologue is in the form of an Excel matrix of 11 500 lines in the following format: “ISO639,^41^ *Language name, Country name, number of L1 speakers, number of L2 speakers”*, along with a large number of related parameters not used for this method, which have been removed.

To obtain the format required by the model (a matrix with all the countries considered in columns and all the languages considered in rows), a series of steps was implemented with the support of different programs written in the form of VBA macros.^42^ One of the most complex steps involved merging all the data from the languages belonging to the same macro-language. This process involved 60 macro-languages comprising 434 different languages: for example, the Arabic macro-language contains 29 languages, such as Egyptian, Arabic, or Moroccan Arabic (see details in Annex 6 of Supplementary material).

After concluding this step, the next process involved reducing the full list of languages to retain only those languages that are handled by the model (number of L1 speakers higher than one million), carefully summing all the remaining numbers by country in a single line for the rest of languages.

It is important to understand that the adoption of Ethnologue data entails the acceptance of its rules of presentation, which are based on purely linguistic considerations:

- Grouping of macro-languages.^43^
- List of countries and corresponding English denominations.

The list of countries treated by Ethnologue is larger than the list treated by the ITU for the provision of Internet connection rates according to country: the ITU, as a United Nations entity, does not separate, for example, Martinique from France. In this case, the ITU rule prevails, and the requirement involves carefully gathering Ethnologue data for the 29 countries not considered by the ITU (for the complete list, see Annex 7 of Supplementary material) into a single column, “Other countries”.^44^

#### 2.5.2. Extrapolation

To overcome the situation of incomplete sources of data per country, the missing values for the undocumented countries must be estimated in the best possible manner. Generally, the missing data is extrapolated from the existing data. Absolute accuracy is not required, but a simple method is needed, of which deviations from reality are real but of limited impact on the results of statistical processing.

Two different methods were adopted to resolve all cases:

a) Extrapolation in proportion to the percentage of people connected by country.

This method will only apply when it is reasonable to consider that the missing values of the micro-indicator values are naturally proportional to the world percentage of people connected (this is the case, for example, for traffic to websites). If the data source is expressed in quantities, the world total must be calculated first. However, if the source is expressed in world percentage, this step will not be necessary. The remaining total or percentage is distributed between the non-documented countries in proportion of their respective weight in terms of the connection to the Internet.

b) Method of quartiles.

In this method, the undocumented countries are filled with a quartile of the source figures depending on their percentage of connected persons. After several tests, it appeared appropriate to determine the allocation of quartiles as follows:

- If <15% of the country's population is connected: the lowest note.
- If more than 15% but <35% of the country's population is connected: first quartile.
- If more than 35% but <65% of the country's population is connected: median.
- If more than 65% but <85% of the country's population is connected: third quartile.
- If more than 85% of the country's population is connected: the highest note.

Typically, micro-indicators, for which no extrapolation method appears obvious, are the same for which the meaning of transforming country figures into language figures does not appear clearly and are thusly excluded.

#### 2.5.3. Source management for micro-indicators

The whole process of managing sources for micro-indicators is the most difficult and demanding task of the project, with a high consumption of human resources. For this purpose, several steps are required:

(a) For each indicator, check that the sources of previous versions are still available and up-to-date, otherwise search for other comparable sources on the Internet.
(b) Select new sources based on their reliability and applicability to the process.^45^
(c) Collect the selected sources in a format allowing for a simplified introduction into the model.
(d) Introduce validated sources into the model.
(e) Assess the source bias.

In Annex 5 of Supplementary material, the complete list of sources is presented, for each indicator.

To perform step (4), the data must be transformed into Excel format, with the country and language names matching those in the template and in the same sequential order.

In step (3), all sources are collected from a specific URL (see Annex 5 of Supplementary material for the complete list of URLs), and most sources are obtained in HTML format. Certain sources are in PDF format, and a limited subset (mainly that of the ITU and the World Bank) is in Excel format, which is the formattargeted to transform all sources. The process of converting from PDF to Excel can be relatively simple in most cases, when the tables are well structured. However, in some cases, an incompatibility exists, and certain tricks are needed, such as going through an intermediate .doc format.

The process of transforming from HTML to Excel can often be a challenging task requiring a lot of imagination, including in certain cases, searching for the data inside the HTML source, and thereafter, trying to build a table using Excel's conversion function after cleaning up the HTML code surrounding the data.

In an increasing number of cases, the source offers geographical access to the data (clickable maps), that, except when the number of countries or languages is limited and manual copying is not cumbersome, makes automated processing impossible or requires the outsourcing of a manual collection work, which is tedious and requires great concentration and discipline to avoid errors.

Credit is due to the institutions (generally, international organizations or NGOs) that report the data in a computer-readable format (Wikimedia provides, for example, in its English version, HTML tables that can be transformed directly into Excel format without loss of structure).

Obtaining a copy of the source in Excel or compatible format (usually a table of country names or languages with associated values or percentages) does not signal the end of the process. With 215 countries and 329 languages to process and, instead of using unambiguous ISO code, the common usage of literal names that can be in different languages and in non-standard spellings, the integration of data into the model cannot be performed by hand. Two programs have been designed for this process, both of which required recursive tuning^46^ to accommodate the different spellings. The program outputs are Excel files that can be used directly to integrate the data into the model. In addition to the appreciable time-saving quality of this computerized method, it guarantees that the obtained data is free of error.

Notably, the management of macro-languages has rendered this process even more complex, because the grouping of languages in the corresponding macro-language must be performed in the source data before processing using the macro. Considering a few examples, the frequent occurrences of Egyptian or Moroccan Arabic in the sources have been cumulated into the Arabic macro-language, whereas those of Serbian, Bosnian, Croatian, and Montenegrin have been merged into Serbo-Croatian (the number of similar cases being quite high). For the manual processing of unknown spellings reported by the program (incorporation of spellings as synonyms or rejection in the other category), the Ethnologue page descriptive of each language code was used in support.^47^

#### 2.5.4. Structure of the model and process

The model is implemented in an Excel file featuring 17 sheets, which are presented in Annex 10 of Supplementary material along with the corresponding process. Notably, database access of these results is scheduled before the end of 2023, with ISO 639-2 codes as the key for access.

## 3. Results

The model generates, for each language, the following indicators, all figures applied for L1+L2:

- (1) Share of the world's L1+L2 speakers.
- (2) Percentage of connected L1+L2 speakers.
- (3) Share of the world's L1+L2 connected speakers.
- (4) Share of total Internet content.
- (5) Virtual presence indicator, defined as the ratio (4)/(1).
- (6) Content Productivity indicator, defined as the ratio (4)/(3).

More elaborated constructions are designed from the aggregation of these indicators, such as those reported in Table 1, as previously mentioned, which offers a global perspective of the situation concerning the different *language families*.^48^ It shows that Asian languages are on their way toward overtaking the European languages, whereas African languages in this regard are lacking, owing to the prevailing digital divide translating into a language divide.^49^

The results of the model can be consulted in CC-BY-SA-4.0 in https://obdilci.org/lc2022 and can be read in Pimienta (2022). Further releases of the model are accessible in https://obdilci.org/Results.

To cross-check the results, the model has been run separately with L1 data only and with L2 data only (see Annex 9 of Supplementary material for the corresponding results, which represent a quite positive indirect control of the method).

## 4. Discussion

The observation of the presence of languages in the Internet has been quite active in the period 2000–2007 (see Pimienta et al., 2009). However, following this period, as mentioned in the Introduction, only two options were available: InternetWorldStats and W3techs.

Both options have presented a highlight of their respective methodology; however, no peer reviewed scientific paper has addressed their respective biases. Their long-term presence without alternative figures have insured them several citations in diverse studies requiring those figures, oftentimes without the necessary caution that would require the reality of their biases.

### 4.1. InternetWorldStats biases

The figures of IWS differ slightly from those of the Observatory, primarily because the sources of demo-linguistic data are not identical, and that, especially for L2 figures, the differences between sources could be considerable (see 2.4.2). However, another difference exists in terms of the management of L2 figures. The Observatory computes the world language percentages for L1+L2 over the number of L1+L2 speakers, a figure 43% greater than the world population, following the Ethnologue source,^50^ whereas IWS computes L1+L2 figures over the world population (named as the zero-sum approach).^51^ Unless there is a trick hidden somewhere in the computations, the zero-sum approach seems to provoke an error by overrating the 10 languages mentioned, error hidden in the remaining languages figure, which will eventually become negative if the number of languages is extended to the point where the sum of L1+L2 speakers crosses the L1 value.

### 4.2. W3Techs biases

The method used by W3Techs involves applying a language-recognition algorithm to the **home page** of 10 million websites that are selected as the most visited sites by certain Web traffic-analysis services (Alexa.com or tranco-list.eu, until the end of 2022).

The differences between W3Techs' and Observatory's figures are immense and generally in a ratio of 1 to 3; sometimes, such as for Chinese and Hindi, this ratio is higher than 1 to 10. Table 4 presents these differences using W3Techs data for 24/8/22 and Observatory data of V3.1 for 8/2022.

**Table 4:** Comparison of figures W3Techs vs. observatory.

	**W3TECHS**		**Observatory**	
**Language**	**Rank**	**Web %** ^a^	**Rank**	**Web %**
English	1	61.4%	1	19.92%
Russian	2	5.6%	4	3,86%
Spanish	3	3.9%	3	8.09%
Turkish	4	3.2%	12	1.15%
German	5	3,1%	10	2.38%
French	6	3.0%	6	3.43%
Persian	7	2.7%	16	0.89%
Chinese	9	1.7%	2	19.82%
Hindi	35	0.1%	5	3.67%

^a^Note that W3Techs offers figure with only one digit after the point.

The highest differences are observed in the values for Hindi and Chinese, and observably, the difference in weight of the English content (over 60% vs. around 20%) raises concern. In August 2022, the statistics aggregator Statista,^52^ based on W3Techs figures, stated that “*English is the Internet's universal language*” while the Observatory concurrently stated that “*The transition of the Internet between the domination of European languages, English in the lead, toward Asian languages and Arabic, Chinese in the lead, is well advanced and the winner is multilingualism, but African languages are slow to take their place”*. Notably, these two statements are not compatible, as at least one statement is invalid.

One could discuss the bias toward English of language recognition algorithms and the bias toward English for selecting the 10 million most visited websites.^53^ However, they are marginal biases that could not explain such huge differences. The main issue lies in the **lack of consideration of multilingualism**, a characteristic of the Web ignored by the W3Techs method which counts a single language for each website, while the Web is probably still more multilingual than humanity.^54^

Considering the background of this discussion, it is important to reiterate the point stated, as documented in Annex 8 of Supplementary material, that Internet users prefer to use their mother tongue in the Net as their first linguistic option and are eager to use their second language(s) in complement.

The problem is rooted in the methodology of measuring **home pages** and counting a single language for each website. Several non-English websites may feature English abstract or few English words in their home pages; thus, these sites are probably counted as English sites. Moreover, many English websites feature several other language versions that will not be counted (as generally observed, if the algorithm is set in an English computed environment, then the website is counted as English only).

The figures yielded by the W3Techs method would be quite different (and hopefully closer to Observatory's figures) if the following rules would be incorporated into its algorithm:

- The counting is performed on webpages but not on websites.
- The algorithm checks the existence of language options in the home page and counts each language offered as an option.
- The algorithm checks the existence of languages other than English in the home page, if this is the case, the website is counted in that language instead of English.
- The algorithm evaluates an approximate number of pages in the website and multiplies each language count with this number after dividing it by the number of language options.

In Pimienta (2023) an attempt is made to un-bias the W3Techs figure for English contents by approximating the rate of multilingualism of the sampling used by W3Tech, Tranco, and from that data establishing the correction to reflect it on the results. It is a simple equation:


$$P^{\prime }=\left(P-Err\right)/Rm,$$

where:

✓ P is the percentage output for English contents in W3Techs
✓ P' is the un-biased percentage for English contents
✓ Err is the percentage of websites erroneously computed as English
✓ Rm is the rate of multilingualism of the sampling.

From the data computed, the range of English contents would slide from the 50%-60% window displayed by W3Techs into the 20–30% windows displayed by the Observatory or the Greek universities study limited to EU ccTLD.

The Observatory have been encouraging the colleagues from Greece to apply their algorithm to the Tranco list of websites, with some promising answer. This would contribute in a definitive manner to that debate since their method does give due credit to the multilingualism of the web. This is an optimistic prospect for the coming months for whoever is interested in that subject.

## 5. Conclusion

For the first time in the Internet's history, a method is able to offer a variety of meaningful indicators concerning the presence of 329 languages on the Internet. The model yields results that are coherent with those of previous studies performed by the Observatory; however, these results are in strong contradiction with those reported by the unique source covering the subject since 2011. In particular, it shows that the English content in the Web today is at the same level as that of the Chinese content, which is around 20%. The fame of W3Techs source have a strong influence on most media which then report the English contents as much above 50% and this situation of misinformation could yield researchers or public policy makers drawing conclusions from good reasoning but on data ground erroneous, and therefore questionable.

The method used to obtain those results is completely and transparently exposed and its biases are openly discussed for further analysis by the scientific community.

These results are simply reflecting a logical step of the evolution of the Web, which evolved from an initial English-centered phase (1992–2000), toward a second step centered in European languages, with English leadership (2000–2010) followed by a more internationalized phase, with the rapid growth of Asian and Arabic languages. Nevertheless, an important gap is leaving the African languages behind, with a Web growing more multilingual everyday (2010–2020). The coming phase (2020–2030) will probably witness a more uniform Web in terms of the representation of languages, with, hopefully, the digital divide starting to break down in Africa, thereby opening the space for the local languages of Africa. The rooting of multilingualism in the Web is underway and may be crossing above that of humanity. Notwithstanding, differences in content productivity will prevail, considering that the certain advantages will benefit some languages with a combination of a large L2 population and country coverage (such as English and French).

The Observatory's figures should not be considered surprising as they simply reflect the natural evolution of the world, reflected in its cyber component. On the contrary, the surprise should stem from the fact that strongly biased figures have been the rule of the last decade, without much reaction from the scientific community.

Hopefully, the full transparency of the method will assist more scientific minds to challenge results pushed by the marketing world and let this theme lie where it should belong: with the scientific community. Clearly, this includes challenging the method exposed hitherto and the detection and discussion of possible biases which have not been adverted by the authors. Let the scientific approach prevail over marketing!

The next release, version 4, along with updating all data sources, will focus on pursuing the bias-reduction effort, especially in the *usage* element, by adding reliable sources for open data and for streaming, and in the *traffic* element, by trying to consider the digital literacy factor.

## Data availability statement

The datasets presented in this study can be found in online repositories. The names of the repository/repositories and accession number(s) can be found below: The model presented stands in 3 type of data: (1) Demo-linguistic data have been bought to Ethnologue and are proprietary with non-disclosure agreement (2) Countries connectivity data are updated yearly by ITU and publicly accessible (3) A large set of data accessible freely on the Internet have been searched and used. Except Ethnologue's all URLs to access input data are provided in the Supplementary material as well as the data output of the model.

## Author contributions

DP is responsible of the conception and the design of the study as well as for the building of the model developed in Excel. ÁB is responsible for the writing of all the computing programs used as resources for the model and for the V3 approach for the traffic indicator. GO is to be credited for linguist support during the study, the coordination with the Brazilian partners, and the writing of part of the introduction. All authors contributed to the article and approved the submitted version.

## Footnotes

^1^Following Ethnologue (https://www.ethnologue.com) the exact number of living languages is 7 168 while other sources compute that around 30 000 languages have existed (https://www.uh.edu/engines/epi2723.htm).

^2^ https://personalpages.manchester.ac.uk/staff/m.dodge/cybergeography/about.html

^3^World Trade Organization (WTO) proposes four modes of service trade: a) from the territory of one Member into the territory of any other Member (Mode 1 - Cross border trade); b)in the territory of one Member to the service consumer of any other Member (Mode 2 - Consumption abroad); c) by a service supplier of one Member, through commercial presence, in the territory of any other Member (Mode 3 - Commercial presence); and 4) by a service supplier of one Member, through the presence of natural persons of a Member in the territory of any other Member (Mode 4 - Presence of natural persons).

^4^ https://www.digitalcommerce360.com/article/global-ecommerce-sales/

^5^ https://w3techs.com/technologies/overview/content_language

^6^ https://w3techs.com/technologies/history_overview/content_language/ms/y

^7^ https://www.internetworldstats.com/stats7.htm

^8^ https://itu.int

^9^ https://www.ethnologue.com/data-consulting

^10^ https://obdilci.org

^11^ https://obdilci.org/lc2022

^12^The method is described in https://funredes.org/lc2017/Alernative%20Languages%20Internet.docx.

^13^ https://en.wikipedia.org/wiki/List_of_Wikipedias

^14^Index refer to rankings in different parameters associated with Information Society progress.

^15^The presence of languages as interface option in a list of applications including on-line translation, as an approximation of a so far non-existing metrics for the level of technological support of languages.

^16^ https://news.netcraft.com/archives/category/web-server-survey

^17^ https://www.worldwidewebsize.com

^18^ https://web.archive.org/web/20011201133152/http://alis.isoc.org/palmares.en.html

^19^ https://obdilci.org/lc2005/english/L1.html

^20^ https://web.archive.org/web/20190904002849/http://maaya.org/?lang=en

^21^ https://tranco-list.eu

^22^APL, “A Programming Language”, which is both a mathematical formalism and its implementation in the form of programming language, designed by Kenneth. Iverson. For more details see https://en.wikipedia.org/wiki/APL(programming_language).

^23^ https://www.itu.int/en/ITU-D/Statistics/Documents/statistics/2021/December/PercentIndividualsUsingInternet.xlsx

^24^ https://data.worldbank.org/indicator/IT.NET.USER.ZS

^25^ http://www.meta-net.eu

^26^A marketing service providing proportion of traffic per country to a large set of websites: https://www.similarweb.com/.

^27^ https://imminent.translated.com/t-index

^28^A simple average without weighting will be used in next release when each element obtains the required sources.

^29^Wikipedia, Wiktionnary, WikiBooks, WikiQuote, WikiVoyage, WikiSources, Wikimedia Commons, WikiSpecies, WikiNews, Wikiversity, and WikiData.

^30^In the two first versions, as *contents* was an input, the outputs indicators were called *Power, Capacity* and *Gradient*, with exactly the same definition as today *Content, Virtual Presence*, and *Content Productivity*.

^31^In terms of %, English + French hold almost 25% of the weight, followed, somehow faraway, by German, Russian, Spanish, and Arabic.

^32^ https://en.wikipedia.org/wiki/List_of_countries_by_English-speaking_population

^33^https://www.statista.com/statistics/266808/the-most-spoken-languages-worldwide/ (real source not cited).

^34^https://www.cambridge.org/core/journals/english-today/article/two-thousand-million/68BFD87E5C867F7C3C47FD0749C7D417.

^35^Note that Alexa has ceased activities in May 2022.

^36^Country Code Top Level Domain.

^37^See the bi-yearly intense conferences and workshops of the LREC researcher's community since 1998: http://www.lrec-conf.org.

^38^Including figures from Facebook, Linkedin, Twitter, Instagram, and Reddit.

^39^See precise definition in https://meta.wikimedia.org/wiki/Wikipedia_article_depth.

^40^ https://funredes.org/lc2021/ALI%20V2-EN.pdf

^41^The 3-character ISO code assigned to each of the 7486 languages identified.

^42^Virtual Basic Applications, a language used to create executable macros in Excel.

^43^A significant example is the case of the Serbo-Croatian macro-language whose definition includes, in alphabetical order, Bosnian, Croatian, Montenegrin, and Serbian. This grouping does not at all meet geopolitical criteria and could even be considered controversial from this point of view. Moreover, as some sources clearly separate the languages and the countries concerned, this entails a risk of error in the results, even if the entry of the sources has been transformed to take this situation into account (the risk arises when the figures must not be summed but rather averaged as in Wikipedia's depth indicator).

^44^It should be noted that Kosovo does have figures reported by the ITU but is absent from Ethnologue's list of countries; consequently, it does not appear in the results.

^45^It may happen that reliable data is in a format that prohibits automated exploitation.

^46^The recursive process recognizes new spellings and ends when the error check no longer identifies unknown spellings.

^47^ https://www.ethnologue.com/language/srp

^48^The language families include, for each region, the languages which are native of that region. English, French, and Spanish are European languages and, following the Ethnologue classification that we use, Russian is classified as European language while Turkish and Hebrew as Asian languages.

^49^Less than 30% of African language's speakers connected to the Internet and very low virtual presence and content productivity are obtained.

^50^In the 2021 figures, those we are using, Ethnologue counts the world population (total number of L1 speakers) at 7 231 699 136 and the total number of L1+L2 speakers at 10 361 716 756.

^51^Cited from the IWS website*: “Indeed, many people are bilingual or multilingual, but here we assign only one language per person in order to have all the language totals add up to the total world population (zero-sum approach)”*.

^52^ https://www.statista.com/chart/26884/languages-on-the-internet/

^53^Following https://news.netcraft.com/archives/category/web-server-survey there are in May 2022 1.16 billion websites of which 270 million are active. The coverage of the most visited is then less than 4% of the total.

^54^It is so if the 270 million active websites offer together more than 400 million different linguistic interfaces, an average in the order of 1.5 by website.
