# Supplementary material for: The method behind the unprecedented production of indicators of the presence of languages in the Internet
Source: Front Res Metr Anal. 2023 May 18;8:1149347. doi: 10.3389/frma.2023.1149347 (PMC10233101; doi:10.3389/frma.2023.1149347)
Supplement: Supplementary file 1 [file Data_Sheet_1.docx]

The method behind the unprecedented production of indicators of the presence of languages in the Internet

Daniel Pimienta^1*^

^1^ Observatory of Linguistic and Cultural Diversity on the Internet, Nice, France

*** Correspondence:**Daniel Pimienta

pimienta@funredes.org

# Supplementary Data

## ANNEX 1: USAGE INDICATOR SOURCES

Table 5: Social networks selected and total subscribers

| **SOCIAL NETWORK** | **TOTAL SUBSCRIBERS**  **(Million)** |
| --- | --- |
| Whatsapp | 2000 |
| Wechat | 1225 |
| Tiktok | 732 |
| Douyin | 600 |
| Telegram | 600 |
| QQ | 595 |
| Snapchat | 528 |
| Weibo | 521 |
| Qzone | 517 |
| Kuaishou | 481 |
| Quora | 300 |
| Skype | 300 |
| Tieba | 300 |
| Viber | 260 |
| IMO | 200 |
| LINE | 169 |
| picsart | 150 |
| Likee | 150 |
| Discord | 140 |
| Twitch | 140 |
| Stack Exchange | 100 |
| VK | 650 |
| Odnoklassniki | 200 |
| Douban | 200 |
| MOJ | 160 |
| JOSH | 115 |
| ShareChat | 160 |
| FACEBOOK %users per country (NapoleonCat 2021) | 1455 |
| INSTAGRAM %users per country (NapoleonCat 2021) | 1200 |
| MESSENGER %users per country (NapoleonCat 2021) | 1300 |
| LINKEDIN %users per country (NapoleonCat 2021) | 155 |
| FACEBOOK World% from IWS 2021 | 1455 |
| Linkedin %user by country (ApolloTech 2021) | 155 |
| Twitter %users per country (Statista 2021) | 396 |
| Pinterest audience % (Statista 2021) | 460 |
| REDDIT % users per country (Statista 2021) | 430 |

Table 6: Sources for social network figures

| SOCIAL NETWORK FIGURES | SOURCE |
| --- | --- |
| FACEBOOK %users per country (NapoleonCat 2021) | https://napoleoncat.com/stats/ |
| INSTAGRAM %users per country (NapoleonCat 2021) | https://napoleoncat.com/stats/ |
| MESSENGER %users per country (NapoleonCat 2021) | https://napoleoncat.com/stats/ |
| LINKEDIN %users per country (NapoleonCat 2021) | https://napoleoncat.com/stats/ |
| Linkedin %user by country (ApolloTech 2021) | https://www.apollotechnical.com/linkedin-users-by-country/ |
| Twitter %users per country (Statista 2021) | https://www.statista.com/statistics/242606/  number-of-active-twitter-users-in-selected-countries/ |
| FACEBOOK World% from IWS 2021 | https://www.internetworldstats.com/stats1.htm + stats2.htm+…stats6.htm |
| Facebook audience % (Statista 2021) | https://www.statista.com/statistics/268136/  top-15-countries-based-on-number-of-facebook-users/ |
| YouTube % of connected within country (Statista 2021) | https://www.statista.com/statistics/1219589/youtube-penetration-worldwide-by-country/ |
| Netflix % subscribers per country (CompariTech 2020) | https://www.comparitech.com/tv-streaming/netflix-subscribers/ |
| Pinterest audience % (Statista 2021) | https://www.statista.com/statistics/328106/pinterest-penetration-markets/ |
| REDDIT % users per country (Statista 2021) | https://backlinko.com/reddit-users |
| Cumul. 2012/21 % OpenOffice downloads per country | http://www.openoffice.org/stats/countries.html |
| # Secure Internet servers | https://data.worldbank.org/indicator/IT.NET.SECR |
| % Fixed broadband subscr. within country (WB 2021) | https://data.worldbank.org/indicator/IT.NET.BBND.P2 |
| % Fixed Tel.+mobile subscr. within country (WB 2021) | https://data.worldbank.org/indicator/IT.MLT.MAIN.P2 +  https://data.worldbank.org/indicator/IT.CEL.SETS.P2 |

## ANNEX 2: ONLINE ENCYCLOPEDIAS ANALYZED FOR CONTENT INDICATOR

Table 7: Online encyclopedias

| LANGUAGE | ENCYCLOPEDIA | NUMBER OF ARTICLES  (Millions) | OTHER INFORMATION |
| --- | --- | --- | --- |
| Various | Encyclopedia of Life ([EOL](http://eol.org)) | 0.75 (2010)  1.9 today | Supported languages: Arabic, Brazilian Portuguese, English, Finnish, French, Macedonian, Piedmontese, Traditional Chinese and Turkish  Interface languages: the same plus German, Spanish, Dutch, Turkish and Ukrainian. |
| Various | thefreedictionary.com/  Free with ads or paid | no statistics | English, Spanish, German, French, Italian, Chinese, Portuguese, Dutch, Norwegian, Greek, Arabic, Polish, Turkish, Russian, Hebrew  It is not clear if they are parallel version or language specific. |
| Various | en.metapedia.org/  neonazi version of wikipedia | Marginal  (5000 articles in English) | Czech, Danish, German, Spanish, English, Hungarian, Dutch, Portuguese, Romanian, Slovenian, Swedish, Estonian, Croatian, Icelandic, Norwegian, Macedonian |
| Chinese | [Baidu Baike](https://baike.baidu.com/) | 24.5 | 194 million edits  7.5 million publishers |
| Chinese | [Baike](https://www.hudong.com) (Hudong) | 18 | 5.8 million publishers (2013) |
| Chinese | [SogouBaike](https://baike.sogou.com) | ????^[[1]](#footnote-1)^ |  |
| Arab | [Marefa](https://www.marefa.org/) | 0.136636 | 2.4 million pages |
| Arab | [Mawdoo3](http://mawdoo3.com) | 0.15 | 45 (2018) |
| Bengali and English | [bengaldia](file:///C:\Users\pimie\AppData\Roaming\Microsoft\Word\en.banglapedia.org) | 0.0057 | 1450 publishers |
| Croatian | enciklopedija.hr | 0.067 | Print version data |
| Croatian | [proleksis.lzmk.hr](http://proleksis.lzmk.hr/) | 0.062 |  |
| Danish | [Den Store Danske](http://denstoredanske.dk/) | 0.161 | 1100 publishers  1 million users |
| Dutch | winklerprins.com | 0.0115 | by subscription |
| English | britannica.com |  | limited free access |
| English | [Everipedia](https://everipedia.org)  Articles copied from wikipedia | ? | 7000 active publishers (2019)  3M users (2017)  free access but also blockchain market ​ |
| English | [Citizenship](https://en.citizendium.org) | 0.017 | Statistics stopped in 2014 close to stopping |
| English | [Conservapedia](https://conservapedia.com) | 0.0518 | 800 million pageviews  1.5 million edits |
| English | [Scholarpedia](http://www.scholarpedia.org) | 0.0018 | Marginal digits |
| English | Encyclopedia.com | 0.3 | Formal encyclopedia aggregator |
| English | [Colombia Encyclopedia](https://en.wikipedia.org/wiki/Columbia_Encyclopedia) |  | Aggregated by Encyclopedia.com |
| English | digitaluniverse.net |  | offline |
| French | [Larousse](https://www.larousse.fr/encyclopedie) | 0.317 |  |
| German | [retro bib](https://www.retrobibliothek.de) | 0.3 |  |
| Hebrew  English | [Hamichlol](file:///C:\Users\pimie\AppData\Roaming\Microsoft\Word\hamichlol.org.il) | 0.28 | Censored version of Wikipedia for a hyper-religious audience |
| Korean | [Doopedia](https://www.doopedia.co.kr) | 0.588 |  |
| Malay Sundanese  Javanese | [Superpedia](http://superpedia.rumahilmu.or.id) | 0.02 |  |
| Italian | [Treccani](https://www.treccani.it/) | 0.9 |  |
| Malayalam | [Sarvavijnanakosam](http://web-edition.sarvavijnanakosam.gov.in/) | 0.007 |  |
| Marathi | [Viswakosh](https://vishwakosh.marathi.gov.in) | 0.016 |  |
| Norwegian Bokmal and Nynorsk | [Store norske leksikon](https://snl.no) | 0.2 (2019) | 3M users/month read 500K articles |
| Polish | encyclopedia.interia.pl | 0.12 (2006) |  |
| Polish | encyclopedia.pwn.pl | 0.08 |  |
| Russian | [Great Russian Encyclopedia](https://bigenc.ru) | 0.012 (2016) |  |
| Russian | [Krugosvet](https://www.krugosvet.ru) | 0.012 |  |
| Spanish | <https://www.ecured.cu/>Cuban | 0.237 | 73,000 active 537 publishers |
| Spanish | [Encyclonet](https://www.enciclonet.com) | 0.185 |  |
| Spanish | enciclopedia.us.es/ | 0.053 | <https://wikiapiary.com/wiki/> |
| Swedish | ne.se/ | 0.26 (2005) |  |
| Tamil | not online |  |  |
| Turkish | [Ekşi Sozluk](https://eksisozluk.com) | 8M admissions in 2009^[[2]](#footnote-2)^ | 400,000 users 110,000 publishers  4M new admissions/year in 2013^[[3]](#footnote-3)^  Open for publication, each entry is kept after moderation. |
| Vietnamese | seems to have disappeared |  | Go to archive.org - https://bachkhoatoanthu.vass.gov.vn |

## ANNEX 3: INTERFACE INDICATOR SOURCES

Table 8: Sources for interface indicator

| Translation languages of Bing Translator | https://www.bing.com/translator/ |
| --- | --- |
| Amazon Kindle direct Publishing supported languages | https://kdp.amazon.com/en_US/help/topic/G200673300 |
| Languages supported by Cortana | https://en.wikipedia.org/wiki/Cortana |
| Word Reference languages supported | https://www.wordreference.com |
| WordLingo Translation languages | http://www.worldlingo.com/en/languages/ |
| Facebook supported languages | https://www.facebook.com/language.php |
| Facebook In-Stream Ads languages supported | https://www.facebook.com/business/help/267128784014981 |
| Free Translator languages supported | http://www.free-translator.com |
| Google Play Console supported languages | https://support.google.com/googleplay/android-developer/table/4419860?hl=en |
| Google Cloud supported languages | https://cloud.google.com/translate/docs/languages?hl=en |
| Google Translate supported languages | https://en.wikipedia.org/wiki/Google_Translate |
| Google Scholar supported languages for search | https://scholar.google.com/scholar_settings?sciifh=1&hl=en&as_sdt=0,5#1 |
| Language supported by Paralink Translator | http://paralink.com |
| Online Translator languages supported | https://www.online-translator.com/traduction |
| Reverso translator languages supported | https://www.reverso.net/text_translation.aspx?lang=EN |
| Free Translation supported languages | https://www.freetranslations.org |
| Skype Supported languages | https://support.skype.com/en/faq/FA34781/what-languages-are-supported-in-skype |
| Systran translate supported languages | https://support.systran.net/systranlinks/faq/ |

## ANNEX 4: INDEX INDICATOR SOURCES

Table 9: Sources for index indicator

| E-Government Index | https://publicadministration.un.org/egovkb/Data-Center |
| --- | --- |
| E-Participation Index | https://publicadministration.un.org/egovkb/Data-Center |
| Online Service Index | https://publicadministration.un.org/egovkb/Data-Center |
| Human Capital Index | https://publicadministration.un.org/egovkb/Data-Center |
| Telecommunication Infrastructure Index | https://publicadministration.un.org/egovkb/Data-Center |
| Cisco Global Digital Readiness Index 2019 | https://www.cisco.com/c/dam/en_us/about/csr/reports/global-digital-readiness-index.pdf |
| Government AI Readiness Index 2020 | https://static1.squarespace.com/static/58b2e92c1e5b6c828058484e/  t/5f7747f29ca3c20ecb598f7c/1601653137399/AI+Readiness+Report.pdf |
| Internet Freedom Scores | https://freedomhouse.org/countries/freedom-net/scores |
| Global Connectivity Index | https://www.huawei.com/minisite/gci/en/country-rankings.html |
| Global Cybersecurity Index 2018 | https://www.itu.int/dms_pub/itu-d/opb/str/D-STR-GCI.01-2018-PDF-E.pdf |
| UNCTAD B2C E-commerce index, 2020 | https://unctad.org/system/files/official-document/tn_unctad_ict4d17_en.pdf |
| The Global Open Data Index | https://index.okfn.org/place/ |
| World Digital Competitiveness Ranking 2020 | https://www.imd.org/globalassets/wcc/docs/release-2020/digital/digital_2020.pdf |
| Readiness For Frontier Technologies Index | https://unctad.org/system/files/official-document/tir2020_en.pdf |
| Global Innovation Index | https://www.wipo.int/edocs/pubdocs/en/wipo_pub_gii_2020.pdf |
| Access to Basic Knowledge | https://legacy.socialprogress.org/assets/downloads/2011-2020-Social-Progress-Index.xlsx |
| Access to Information and Communications | https://legacy.socialprogress.org/assets/downloads/2011-2020-Social-Progress-Index.xlsx |
| Access to Advanced Education | https://legacy.socialprogress.org/assets/downloads/2011-2020-Social-Progress-Index.xlsx |
| Access to electricity (% of pop.) | https://legacy.socialprogress.org/assets/downloads/2011-2020-Social-Progress-Index.xlsx |
| Access to quality education (0=unequal; 4=equal) | https://legacy.socialprogress.org/assets/downloads/2011-2020-Social-Progress-Index.xlsx |
| Access to online governance (0=low; 1=high) | https://legacy.socialprogress.org/assets/downloads/2011-2020-Social-Progress-Index.xlsx |
| Media censorship (0=frequent; 4=rare) | https://legacy.socialprogress.org/assets/downloads/2011-2020-Social-Progress-Index.xlsx |
| Freedom of expression (0=no freedom; 1=full freedom) | https://legacy.socialprogress.org/assets/downloads/2011-2020-Social-Progress-Index.xlsx |
| Quality weighted universities (points) | https://legacy.socialprogress.org/assets/downloads/2011-2020-Social-Progress-Index.xlsx |
| Citable documents | https://legacy.socialprogress.org/assets/downloads/2011-2020-Social-Progress-Index.xlsx |
| Women with advanced education | https://legacy.socialprogress.org/assets/downloads/2011-2020-Social-Progress-Index.xlsx |
| Years of tertiary schooling | https://legacy.socialprogress.org/assets/downloads/2011-2020-Social-Progress-Index.xlsx |

## ANNEX 5: TRAFFIC INDICATOR WEBSITES SELECTION

Table 10: Selection of websites for traffic indicator

| **WEBSITE** | **NUMBER OF TIMES** |
| --- | --- |
| 10086.cn | 1 |
| 10jqka.com.cn | 1 |
| 122.gov.cn | 1 |
| 12306.cn | 1 |
| 12371.cn | 1 |
| 12377.cn | 1 |
| 12388.gov.cn | 1 |
| 1688.com | 1 |
| 17ok.com | 1 |
| 189.cn | 1 |
| 1c-bitrix.ru | 1 |
| 22.cn | 1 |
| 24.kg | 1 |
| 24h.com.vn | 1 |
| 2gis.ru | 1 |
| 300.cn | 1 |
| 360.cn | 1 |
| 4.cn | 1 |
| 6.cn | 1 |
| 66law.cn | 1 |
| 999.md | 1 |
| abc.com.py | 1 |
| abc-communication.dz | 1 |
| abril.com.br | 1 |
| accuweather.com | 2 |
| activemind.de | 1 |
| actuniger.com | 1 |
| ad.iq | 1 |
| admin.ch | 1 |
| adminbuy.cn | 1 |
| Adobe.com | 1 |
| afrikmag.com | 1 |
| agenziaentrate.gov.it | 1 |
| ah.gov.cn | 1 |
| ahoraeg.com | 1 |
| ahram.org.eg | 1 |
| aktuality.sk | 1 |
| alakhbar.info | 1 |
| aliexpress.com | 1 |
| alipay.com | 1 |
| allegro.pl | 1 |
| allevents.in | 1 |
| almasryalyoum.com | 1 |
| alraziuni.edu.ye | 1 |
| alwakeelnews.com | 1 |
| alwatanvoice.com | 1 |
| amazon.ae | 1 |
| amazon.ca | 1 |
| amazon.cn | 1 |
| amazon.co.jp | 1 |
| amazon.co.uk | 1 |
| amazon.com | 20 |
| amazon.com.br | 1 |
| amazon.de | 1 |
| amazon.eg | 1 |
| amazon.es | 1 |
| amazon.fr | 1 |
| amazon.in | 1 |
| amazon.it | 1 |
| ameblo.jp | 1 |
| amritmahotsav.nic.in | 1 |
| andersnoren.se | 1 |
| anpc.gov.ro | 1 |
| anyxxx.com | 1 |
| ap.gov.in | 1 |
| aparat.com | 2 |
| apple.com | 5 |
| arabiaweather.com | 1 |
| argentina.gob.ar | 1 |
| aruba.it | 1 |
| autohome.com.cn | 1 |
| avaz.ba | 1 |
| babytree.com | 1 |
| baharain.bh | 1 |
| baidu.com | 4 |
| band.us | 1 |
| bangladesh.gov.bd | 1 |
| bankmandiri.co.id | 1 |
| bayern.de | 1 |
| bb.com.br | 1 |
| bbc.co.uk | 1 |
| bbc.com | 1 |
| bbc.in | 1 |
| bcel.com.la | 1 |
| beian.gov.cn | 1 |
| beijing.gov.cn | 1 |
| belgium.be | 1 |
| belizebank.com | 1 |
| belonnanotservice.ga | 2 |
| bet365.com | 1 |
| bgeneral.com | 1 |
| bih.nic.in | 1 |
| Bing.com | 3 |
| biobiochile.cl | 1 |
| bitrix24.ru | 1 |
| bjx.com.cn | 1 |
| blogger.com | 3 |
| bnonline.fi.cr | 1 |
| boc.cn | 1 |
| Bongacams.com | 3 |
| borneobulletin.com.bn | 1 |
| bri.co.id | 1 |
| britannica.com | 2 |
| bshare.cn | 1 |
| bt.bt | 1 |
| bt.cn | 1 |
| bukalapak.com | 1 |
| bund.de | 1 |
| businessday.ng | 1 |
| businessinsider.in | 1 |
| businesstoday.in | 1 |
| businessworld.in | 1 |
| cac.gov.cn | 1 |
| cafebazaar.ir | 1 |
| caixa.gov.br | 1 |
| cambridge.org | 1 |
| Canva.com | 5 |
| cao.ir | 1 |
| careers.sl | 1 |
| cas.cn | 1 |
| cbec.gov.in | 1 |
| cbic.gov.in | 1 |
| cbos.gov.sd | 1 |
| cbse.gov.in | 1 |
| cbse.nic.in | 1 |
| ccdi.gov.cn | 1 |
| ccgp.gov.cn | 1 |
| ccm.gov.cn | 1 |
| ce.cn | 1 |
| centrafrique-presse.over-blog.com | 1 |
| chase.com | 1 |
| chaturbate.com | 2 |
| china.cn | 1 |
| china.com.cn | 1 |
| chinadaily.com.cn | 1 |
| chinanews.com.cn | 1 |
| chinatax.gov.cn | 1 |
| chsi.com.cn | 1 |
| cib.com.cn | 1 |
| cmbc.com.cn | 1 |
| cmseasy.cn | 1 |
| cnil.fr | 1 |
| cninfo.com.cn | 1 |
| cnipa.gov.cn | 1 |
| cnnindonesia.com | 1 |
| cnpq.br | 1 |
| cnr.cn | 1 |
| cntv.cn | 1 |
| coinmarketcap.com | 2 |
| comores-infos.net | 1 |
| conac.cn | 1 |
| consultant.ru | 1 |
| coremail.cn | 1 |
| correios.com.br | 1 |
| corriere.it | 1 |
| coupa.ng | 1 |
| court.gov.cn | 1 |
| covid19.go.id | 1 |
| cowin.gov.in | 1 |
| cpdp.bg | 1 |
| cq.gov.cn | 1 |
| creditchina.gov.cn | 1 |
| cri.cn | 1 |
| cricbuzz.com | 1 |
| cro.ma | 1 |
| csdn.net | 1 |
| csrc.gov.cn | 1 |
| customs.gov.cn | 1 |
| cvc.nic.in | 1 |
| cyberpolice.cn | 1 |
| dahe.cn | 1 |
| dailypost.ng | 1 |
| dakaractu.com | 1 |
| daraz.pk | 1 |
| data.gov.in | 1 |
| dataprotection.gov.cy | 1 |
| daum.net | 1 |
| defimedia.info | 1 |
| detik.com | 1 |
| dg-datenschutz.de | 1 |
| dictionary.com | 1 |
| digikala.com | 1 |
| digitalindia.gov.in | 1 |
| dinesh-ghimire.com.np | 1 |
| discord.com | 1 |
| ditaduraeconsenso.blogspot.com | 1 |
| dlszywz.cn | 1 |
| dns4.cn | 1 |
| docdro.id | 1 |
| dpboss.net | 1 |
| dr.dk | 1 |
| draugiem.lv | 1 |
| duckduckgo.com | 1 |
| dwz.cn | 1 |
| e.gov.kw | 1 |
| ebay.com | 1 |
| ebay.de | 1 |
| ebs.org.cn | 1 |
| eci.gov.in | 1 |
| education.gov.in | 1 |
| elcomercio.com | 1 |
| eldeber.com.bo | 1 |
| elnuevodia.com | 1 |
| elpais.com | 1 |
| elsalvador.com | 1 |
| eluniverso.com | 1 |
| emansion.gov.lr | 1 |
| emploi.cg | 1 |
| ems.com.cn | 1 |
| enamad.ir | 1 |
| enimerotiko.gr | 1 |
| eol.cn | 1 |
| e-recht24.de | 1 |
| ernet.in | 1 |
| espn.com | 1 |
| espncricinfo.com | 1 |
| estadao.com.br | 1 |
| eta.gov.lk | 1 |
| ethiojobs.net | 1 |
| etnet.com.hk | 1 |
| facebook.com | 80 |
| facebook.com.br | 1 |
| fandom.com | 3 |
| fazenda.gov.br | 1 |
| fijivillage.com | 1 |
| file-upload.com | 1 |
| findlaw.cn | 1 |
| firefox.com.cn | 1 |
| fiverr.com | 1 |
| flipkart.com | 1 |
| flydubai.com | 1 |
| fmprc.gov.cn | 1 |
| focus.cn | 1 |
| follow.it | 1 |
| Force.com | 1 |
| free.fr | 1 |
| freebitco.in | 1 |
| freeindianporn2.com | 1 |
| freepik.com | 1 |
| fs.fed.us | 1 |
| ftc.go.kr | 1 |
| fujian.gov.cn | 1 |
| gansu.gov.cn | 1 |
| garanteprivacy.it | 1 |
| gd.gov.cn | 1 |
| geni.us | 1 |
| gesetze-im-internet.de | 1 |
| ghanaweb.com | 1 |
| gismeteo.ru | 1 |
| globo.com | 1 |
| gmw.cn | 1 |
| gogo.mn | 1 |
| gome.com.cn | 1 |
| goo.ne.jp | 1 |
| google,com | 1 |
| google.ad | 1 |
| google.ae | 1 |
| google.at | 1 |
| google.az | 1 |
| google.be | 1 |
| google.bf | 1 |
| google.bg | 1 |
| google.ca | 2 |
| google.cd | 1 |
| google.cg | 1 |
| google.ch | 1 |
| google.ci | 1 |
| google.cl | 1 |
| google.cn | 1 |
| google.co.id | 1 |
| google.co.il | 1 |
| google.co.in | 1 |
| google.co.jp | 1 |
| google.co.ke | 1 |
| google.co.kr | 1 |
| google.co.ma | 1 |
| google.co.mz | 1 |
| google.co.nz | 1 |
| google.co.th | 1 |
| google.co.tz | 1 |
| google.co.ug | 1 |
| google.co.uk | 1 |
| google.co.uz | 1 |
| google.co.ve | 1 |
| google.co.za | 1 |
| google.co.zm | 1 |
| google.co.zw | 1 |
| google.com | 146 |
| google.com.af | 1 |
| google.com.ar | 1 |
| google.com.bd | 1 |
| google.com.bn | 1 |
| google.com.bo | 1 |
| google.com.br | 1 |
| google.com.bz | 1 |
| google.com.co | 1 |
| google.com.cu | 1 |
| google.com.do | 1 |
| google.com.eg | 1 |
| google.com.hk | 2 |
| google.com.jm | 1 |
| google.com.kw | 1 |
| google.com.lb | 1 |
| google.com.ly | 1 |
| google.com.mm | 1 |
| google.com.mt | 1 |
| google.com.mx | 1 |
| google.com.na | 1 |
| google.com.ng | 1 |
| google.com.ni | 1 |
| google.com.np | 1 |
| google.com.om | 1 |
| google.com.pa | 1 |
| google.com.pe | 1 |
| google.com.pg | 1 |
| google.com.ph | 1 |
| google.com.pk | 1 |
| google.com.pr | 1 |
| google.com.py | 1 |
| google.com.qa | 1 |
| google.com.sa | 1 |
| google.com.sb | 1 |
| google.com.sg | 1 |
| google.com.sl | 1 |
| google.com.sv | 1 |
| google.com.tj | 1 |
| google.com.tr | 1 |
| google.com.tw | 1 |
| google.com.ua | 1 |
| google.com.uy | 1 |
| google.com.vn | 1 |
| google.de | 1 |
| google.dj | 1 |
| google.dk | 1 |
| google.dz | 1 |
| google.ee | 1 |
| google.es | 2 |
| google.fr | 3 |
| google.ge | 1 |
| google.gr | 1 |
| google.gy | 1 |
| google.hn | 1 |
| google.ht | 1 |
| google.ie | 1 |
| google.iq | 1 |
| google.is | 1 |
| google.it | 1 |
| google.jo | 1 |
| google.kg | 1 |
| google.kz | 1 |
| google.la | 1 |
| google.lk | 1 |
| google.lt | 1 |
| google.lu | 1 |
| google.lv | 1 |
| google.md | 1 |
| google.me | 1 |
| google.mg | 1 |
| google.mk | 1 |
| google.ml | 1 |
| google.mn | 1 |
| google.mw | 2 |
| google.nl | 1 |
| google.no | 1 |
| google.pl | 1 |
| google.ps | 1 |
| google.pt | 1 |
| google.ro | 1 |
| google.rs | 1 |
| Google.ru | 3 |
| google.rw | 1 |
| google.se | 1 |
| google.si | 1 |
| google.sk | 1 |
| google.sn | 1 |
| google.so | 1 |
| google.sr | 1 |
| google.st | 1 |
| google.td | 1 |
| google.tg | 1 |
| google.tl | 1 |
| google.tm | 1 |
| google.tn | 1 |
| google.tt | 1 |
| gosuslugi.ru | 1 |
| gov.bw | 1 |
| gov.ls | 1 |
| govtrack.us | 1 |
| grid.id | 1 |
| grupobancolombia.com | 1 |
| gst.gov.in | 1 |
| gsxt.gov.cn | 1 |
| guardian.co.tt | 1 |
| guardian.ng | 1 |
| gujarat.gov.in | 1 |
| gxzf.gov.cn | 1 |
| gz.gov.cn | 1 |
| haberler.com | 1 |
| hainan.gov.cn | 1 |
| haosou.com | 1 |
| hatena.ne.jp | 1 |
| hd315.gov.cn | 1 |
| hdfcbank.com | 1 |
| healthline.com | 1 |
| heartland.us | 1 |
| henan.gov.cn | 1 |
| herald.co.zw | 1 |
| hi.is | 1 |
| hindustantimes.com | 1 |
| homedepot.com | 1 |
| hoster.kz | 1 |
| hotlog.ru | 1 |
| hotpepper.jp | 1 |
| hotstar.com | 2 |
| huanqiu.com | 1 |
| hubei.gov.cn | 1 |
| hunan.gov.cn | 1 |
| hurriyet.com.tr | 1 |
| ibps.in | 1 |
| ibw.cn | 1 |
| icbc.com.cn | 1 |
| icicibank.com | 1 |
| icio.us | 1 |
| ico.org.uk | 1 |
| idnes.cz | 1 |
| iitb.ac.in | 1 |
| iitkgp.ac.in | 1 |
| ijavhd.com | 1 |
| imageshack.us | 1 |
| imdb.com | 2 |
| imjo.in | 1 |
| in.gr | 1 |
| incometax.gov.in | 1 |
| incometaxindia.gov.in | 1 |
| incometaxindiaefiling.gov.in | 1 |
| index.hr | 1 |
| index.hu | 1 |
| india.com | 1 |
| india.gov.in | 1 |
| indiamart.com | 1 |
| indianrailways.gov.in | 1 |
| indianvisaonline.gov.in | 1 |
| indiapost.gov.in | 1 |
| indiatimes.com | 1 |
| indiatoday.in | 1 |
| inflibnet.ac.in | 1 |
| instagram.com | 47 |
| instructure.com | 1 |
| intoday.in | 1 |
| iol.co.za | 1 |
| ionos.de | 1 |
| iplt20.com | 1 |
| irctc.co.in | 1 |
| irembo.gov.rw | 1 |
| irna.ir | 1 |
| is.fi | 1 |
| isna.ir | 1 |
| itau.com.br | 1 |
| jamaica-gleaner.com | 1 |
| japanpost.jp | 1 |
| jc001.cn | 1 |
| Jd.com | 1 |
| jiangsu.gov.cn | 1 |
| jiangxi.gov.cn | 1 |
| jiji.ng | 1 |
| jl.gov.cn | 1 |
| jne.co.id | 1 |
| jotform.us | 1 |
| jrj.com.cn | 1 |
| jumia.ci | 1 |
| jumia.com.ng | 1 |
| juraforum.de | 1 |
| justindianporn.me | 1 |
| kancloud.cn | 1 |
| kar.nic.in | 1 |
| karnataka.gov.in | 1 |
| kaskus.co.id | 1 |
| kemdikbud.go.id | 1 |
| kemenag.go.id | 1 |
| kemkes.go.id | 1 |
| kenh14.vn | 1 |
| kerala.gov.in | 1 |
| khaberni.com | 1 |
| knet.cn | 1 |
| knetreg.cn | 1 |
| kominfo.go.id | 1 |
| kompas.com | 1 |
| kriesi.at | 1 |
| kuaishang.cn | 1 |
| kuenselonline.com | 1 |
| kumparan.com | 1 |
| kupujemprodajem.com | 1 |
| lanouvelletribune.info | 1 |
| laodong.vn | 1 |
| laprensa.com.ni | 1 |
| laprensa.hn | 1 |
| lawtime.cn | 1 |
| lazada.co.id | 1 |
| leader.ir | 1 |
| lefigaro.fr | 1 |
| legifrance.gouv.fr | 1 |
| legit.ng | 1 |
| lemonde.fr | 1 |
| lex.uz | 1 |
| licindia.in | 1 |
| line.me | 2 |
| linkd.in | 1 |
| linkedin.com | 13 |
| liputan6.com | 1 |
| list.am | 1 |
| listindiario.com | 1 |
| live.com | 19 |
| liveinternet.ru | 1 |
| livroreclamacoes.pt | 1 |
| lnkd.in | 1 |
| ltn.com.tw | 1 |
| ltt.ly | 1 |
| m.in | 1 |
| macaodaily.com | 1 |
| mahaonline.gov.in | 1 |
| maharashtra.gov.in | 1 |
| mail.ru | 2 |
| mana.pf | 1 |
| mastercard.us | 1 |
| mayoclinic.org | 2 |
| medcol.mw | 1 |
| mediacongo.net | 1 |
| mercadolibre.cl | 1 |
| mercadolibre.com.co | 1 |
| mercadolibre.com.ve | 1 |
| mercadolivre.com.br | 1 |
| merdeka.com | 1 |
| merriam-webster.com | 1 |
| meskerem.net | 1 |
| meteo.nc | 1 |
| metruyenchu.com | 1 |
| mhlw.go.jp | 1 |
| microsoft.com | 25 |
| microsoftonline.com | 4 |
| milliyet.com.tr | 1 |
| mk.by | 1 |
| mof.gov.tl | 1 |
| moh.go.tz | 1 |
| moip.gov.mm | 1 |
| mol.gov.om | 1 |
| monetizze.com.br | 1 |
| msn.com | 3 |
| myshopify.com | 5 |
| namibian.com.na | 1 |
| namnak.com | 1 |
| naver.com | 1 |
| ncdc.gov.ng | 1 |
| nessma.tv | 1 |
| netafrique.net | 1 |
| netflix.com | 13 |
| nethouse.ru | 1 |
| nettruyengo.com | 1 |
| news24.com | 1 |
| niagahoster.co.id | 1 |
| notion.so | 1 |
| novinky.cz | 1 |
| nsw.gov.au | 1 |
| nzherald.co.nz | 1 |
| odnoklassniki.ru | 1 |
| office.com | 8 |
| ok.ru | 3 |
| okezone.com | 1 |
| onlinehome.us | 1 |
| orange.fr | 1 |
| orient.tm | 1 |
| otr.tg | 1 |
| ouest-france.fr | 1 |
| oxu.az | 1 |
| ozon.ru | 1 |
| pagcor.ph | 1 |
| pagesjaunes.fr | 1 |
| paypal.com | 1 |
| paystack.com | 1 |
| pikiran-rakyat.com | 1 |
| pinterest.com | 11 |
| pinterest.de | 1 |
| pinterest.es | 1 |
| pinterest.fr | 1 |
| pinterest.it | 1 |
| pixnet.net | 1 |
| planalto.gov.br | 1 |
| pornhub.com | 4 |
| portaldoconhecimento.gov.cv | 1 |
| post.ir | 1 |
| postcourier.com.pg | 1 |
| postimees.ee | 1 |
| premierbet.co.ao | 1 |
| premierleague.com | 1 |
| prensa-latina.cu | 1 |
| prensalibre.com | 1 |
| presidence.gov.bi | 1 |
| president.ir | 1 |
| president.tj | 1 |
| prom.st | 1 |
| prom.ua | 1 |
| public.lu | 1 |
| pulse.ng | 1 |
| punchng.com | 1 |
| qq.com | 2 |
| r01.ru | 1 |
| rae.es | 1 |
| rakuten.co.jp | 1 |
| rambler.ru | 1 |
| reddit.com | 9 |
| reg.ru | 1 |
| repubblica.it | 1 |
| republika.co.id | 1 |
| ria.ru | 1 |
| rijksoverheid.nl | 1 |
| rt.com | 1 |
| rte.ie | 1 |
| rtvslo.si | 1 |
| s.id | 1 |
| sabay.com.kh | 1 |
| sacoronavirus.co.za | 1 |
| sahibinden.com | 1 |
| sakura.ne.jp | 1 |
| salesforce.com | 1 |
| salla.sa | 1 |
| sana.sy | 1 |
| sante.gov.dz | 1 |
| sante.gov.gn | 1 |
| sapo.pt | 1 |
| sapp.ir | 1 |
| saude.gov.br | 1 |
| scielo.br | 1 |
| sekolahku.web.id | 1 |
| seneweb.com | 1 |
| serveriai.lt | 1 |
| service-public.fr | 1 |
| setn.com | 1 |
| seznam.cz | 1 |
| shopee.co.id | 1 |
| shopee.co.th | 1 |
| shopee.tw | 1 |
| shopee.vn | 1 |
| shop-pro.jp | 1 |
| singaporepools.com.sg | 1 |
| smarturl.it | 1 |
| sohu.com | 2 |
| solomonstarnews.com | 1 |
| soy502.com | 1 |
| spiegel.de | 1 |
| stackoverflow.com | 1 |
| standardmedia.co.ke | 1 |
| state.co.us | 1 |
| state.fl.us | 1 |
| state.il.us | 1 |
| state.ma.us | 1 |
| state.md.us | 1 |
| state.mn.us | 1 |
| state.nj.us | 1 |
| state.nm.us | 1 |
| state.nv.us | 1 |
| state.ny.us | 1 |
| state.oh.us | 1 |
| state.or.us | 1 |
| state.pa.us | 1 |
| state.tx.us | 1 |
| suara.com | 1 |
| sucursalelectronica.com | 1 |
| suribet.sr | 1 |
| sympla.com.br | 1 |
| syri.net | 1 |
| t.me | 2 |
| taobao.com | 2 |
| theguardian.com | 1 |
| thethao247.vn | 1 |
| tiktok.com | 10 |
| time.mk | 1 |
| times.co.sz | 1 |
| timesofmalta.com | 1 |
| timeweb.ru | 1 |
| tmall.com | 1 |
| tokopedia.com | 1 |
| t-online.de | 1 |
| tradingview.com | 2 |
| trendyol.com | 1 |
| tribunnews.com | 1 |
| tripadvisor.com.br | 1 |
| tripadvisor.fr | 1 |
| tripadvisor.it | 1 |
| turkiye.gov.tr | 1 |
| twitch.tv | 5 |
| twitter.com | 32 |
| ucoz.ru | 1 |
| uem.mz | 1 |
| ultimahora.com | 1 |
| uol.com.br | 1 |
| ura.go.ug | 1 |
| usp.br | 1 |
| vanguardngr.com | 1 |
| vg.no | 1 |
| vk.com | 7 |
| vkontakte.ru | 1 |
| vnexpress.net | 1 |
| walmart.com | 1 |
| wbs-law.de | 1 |
| weather.com | 1 |
| webmd.com | 1 |
| whatsapp.com | 22 |
| wikipedia.org | 29 |
| wiktionary.org | 2 |
| wildberries.ru | 1 |
| wizard.id | 1 |
| www.gob.mx | 1 |
| www.gob.pe | 1 |
| www.gov.br | 1 |
| www.gov.pl | 1 |
| www.gov.uk | 1 |
| xhamster.com | 1 |
| xnxx.com | 3 |
| xosodaiphat.com | 1 |
| xvideos.com | 6 |
| yahoo.co.jp | 1 |
| yahoo.com | 25 |
| yandex.ru | 5 |
| yasour.org | 1 |
| yelp.com | 1 |
| ynet.co.il | 1 |
| youm7.com | 1 |
| youtu.be | 1 |
| youtube.com | 103 |
| youtube.org | 1 |
| zalo.me | 1 |
| zambiaimmigration.gov.zm | 1 |
| zhizhuchi.cm | 1 |
| zoom.us | 15 |

## ANNEX 6: MACROLANGUAGES

As defined by Ethnologue.

Table 11: List of macro-languages

| ***ISO CODE*** | ***MACRO***  ***LANGUAGES*** | ***NUMBER OF LANGUAGES***  ***MERGED*** |
| --- | --- | --- |
| *ara* | **Arabic** | ***29*** |
| *aym* | **Aymara** | ***2*** |
| *aze* | **Azerbaijani** | ***3*** |
| *bal* | **Baluchi** | ***3*** |
| *bik* | **Bikol** | ***8*** |
| *bnc* | **Bontok** | ***5*** |
| *bua* | **Buryat** | ***3*** |
| *chm* | **Mari** | ***2*** |
| *cre* | **Cree** | ***6*** |
| *del* | **Delaware** | ***2*** |
| *den* | **Slave** | ***2*** |
| *din* | **Dinka** | ***5*** |
| *doi* | **Dogri** | ***2*** |
| *est* | **Estonian** | ***2*** |
| *fas* | **Persian** | ***2*** |
| *ful* | **Fulfulde** | ***9*** |
| *gba* | **Gbaya** | ***6*** |
| *gon* | **Gondi** | ***3*** |
| *grb* | **Grebo** | ***5*** |
| *grn* | **Guarani** | ***5*** |
| *hai* | **Haida** | ***2*** |
| *hbs* | **Serbo-croatian** | ***4*** |
| *hmn* | **Hmong** | ***25*** |
| *iku* | **Inuktitut** | ***2*** |
| *ipk* | **Inupiatun** | ***2*** |
| *jrb* | **Judeo-arabic** | ***5*** |
| *kau* | **Kanuri** | ***3*** |
| *kln* | **Kalenjin** | ***9*** |
| *kok* | **Konkani** | ***2*** |
| *kom* | **Komis** | ***2*** |
| *kon* | **Congo** | ***3*** |
| kpe | **Kpell** | **2** |
| *kur* | **Kurdish** | ***3*** |
| *lah* | **Lahnda** | ***7*** |
| *lav* | **Latvian** | ***2*** |
| *luy* | **Luiya** | ***14*** |
| *man* | **Mandingo** | ***6*** |
| *mlg* | **Malagasy** | ***11*** |
| *mon* | **Mongolian** | ***3*** |
| *msa* | **Malay** | ***36*** |
| *mwr* | **Marwari** | ***6*** |
| *nep* | **Nepalese** | ***2*** |
| *oji* | **Ojibway** | ***7*** |
| *ori* | **Oriya** | ***2*** |
| *orm* | **Galla** | ***4*** |
| *pus* | **Pashto** | ***3*** |
| *que* | **Quechua** | ***42*** |
| *raj* | **Rajasthan** | **6** |
| *rom* | **Romani** | ***6*** |
| *sqi* | **Albanian** | ***4*** |
| *srd* | **Sardinian** | ***4*** |
| *swa* | **Swahili** | ***2*** |
| *syr* | **Syriac** | ***2*** |
| *tmh* | **Tamashek** | ***4*** |
| *uzb* | **Uzbek** | ***2*** |
| *yid* | **Yiddish** | ***2*** |
| *zap* | **Zapotec** | ***57*** |
| *zha* | **Zhuang** | ***16*** |
| *zho* | **Chinese** | ***15*** |
| *zza* | **Dimli** | ***2*** |

## ANNEX 7: LIST OF COUNTRIES OR TERRITORIES WITH NO ITU DATA

Table 12: List of countries with no ITU data

| **ISO code** | **NAME OF THE COUNTRY** | **POPULATION** |
| --- | --- | --- |
| **AX** | **Åland Island** | **27,652** |
| **AS** | **American Samoa** | **55,990** |
| **IO** | **British Indian Ocean Territory** | **4,000** |
| **QB** | **Caribbean Netherlands** | **18,740** |
| **CX** | **Christmas Island** | **1,170** |
| **CC** | **Cocos (Keeling) Islands** | **630** |
| **CK** | **Cook Islands** | **15,000** |
| **CW** | **Curacao** | **140,000** |
| **GF** | **French Guiana** | **366,590** |
| **GP** | **Guadeloupe** | **454,800** |
| **GU** | **Guam** | **139,550** |
| **IM** | **Isle of man** | **88,085** |
| **QM** | **Martinique** | **377 100** |
| **NC** | **Norfolk Island** | **1,500** |
| ***KP*** | ***North Korea*** | ***25,579,000*** |
| **PM** | **Northern Mariana Islands** | **53,280** |
| **PW** | **Palau** | **17,550** |
| **PN** | **Pitcairn** | **36** |
| **RE** | **Réunion** | **751,580** |
| **BL** | **Saint Barthélemy** | **7,850** |
| **FM** | **Saint-Martin** | **28,500** |
| **PM** | **Saint Pierre and Miquelon** | **6,340** |
| **SX** | **Saint-Martin** | **33,470** |
| **CT** | **Turks and Caicos Islands** | **30 170** |
| ***GO*** | ***Vatican State*** | ***330*** |
| ***HE*** | ***Western Sahara*** | ***544 150*** |
|  | **TOTAL** | **28,689,463** |

There are two possible reasons why the country or territory is excluded from ITU data:

- It is a territory whose data is included in a given country
- There is no source or estimate of the percentage of people connected to the Internet (in italics in the table).

## ANNEX 8 : SOURCES ABOUT LANGUAGE BEHAVIOR OF INTERNAUTS

<https://motsdici.be/wp-content/uploads/2019/04/Article-cant-read-wont-buy.pdf>

2006 Common Sense Advisory Report

<https://ec.europa.eu/commission/presscorner/detail/en/IP_11_556>

2011 Union European Survey Report “*Digital Agenda: more than half EU Internet surfers use foreign language when online*”

Citation : *“While 90% of Internet surfers in the EU prefer to access websites in their own language, 55% at least occasionally use a language other than their own when online according to a pan-EU Eurobarometer”.*

<https://hbr.org/2012/08/speak-to-global-customers-in-t>

2012 Harvard Business Review*: “Speak to Global Customers in Their Own Language”*

Citation: *“72.1% of consumers spend most or all of their time on websites in their own language”*

<https://assets.kpmg/content/dam/kpmg/in/pdf/2017/04/Indian-languages-Defining-Indias-Internet.pdf>

2017 KPMG/Google study “*Indian languages – Defining India’s Internet*”

Citation : “*Indian languages Internet users are expected to account for nearly 75% of Indias’s Internet user base in 2021.*”

<https://insights.csa-research.com/reportaction/305013126/Marketing>

<https://csa-research.com/Blogs-Events/CSA-in-the-Media/Press-Releases/Consumers-Prefer-their-Own-Language>

2020 CSA Research Report *“Can’t Read, Won’t Buy – B2C Analyzing Consumer Language Preferences and Behaviors in 29 Countries”*

Citation: *Survey of 8,709 Consumers in 29 Countries Finds that 76% Prefer Purchasing Products with Information in their Own Language*

<https://octopustranslations.com/e-commerce-and-the-impact-of-language-on-consumer-behavior/>

2021 Octopus Translation report *E-commerce and the Impact of Language on Consumer Behavior*

Citation: *55% of consumers around the world make their purchases online only in their mother tongue*

<https://www.businesswire.com/news/home/20211026005375/en/Unbabel%E2%80%99s-2021-Global-Multilingual-CX-Survey-Reveals-68-of-Consumers-Prefer-to-Speak-with-Brands-in-Their-Native-Language>

2021 BusinessWire Report “*Unbabel’s 2021 Global Multilingual CX Survey Reveals 68% of Consumers Prefer to Speak with Brands in Their Native Language”*

<https://www.prweb.com/releases/2014/04/prweb11725995.htm>

*2022.* PRWeb Market Research *“Survey of 3,000 Online Shoppers across 10 Countries Finds that 60% Rarely or Never Buy from English-only Websites”*

## ANNEX 9: SEPARATE MODEL RUN FOR L1 AND L2

As a method for cross-checking the validity of the model, which is based on L1+L2 demo-linguistic data, two additional runs were made, one with L1 figures only and another one with L2 figures only.

Table 13: Model run with L1 only

|  |  | **Internet users** | **Population** | **Speakers** | **Contents** | **Presence** | **Productivity** |
| --- | --- | --- | --- | --- | --- | --- | --- |
| **L1** |  | **L1** | **L1** | **Connected** | **L1** | **Virtual** | **Contents** |
| 1 | Chinese | 22.34% | 18.33% | 71.18% | **25.55%** | **1.39** | **1.14** |
| 2 | English | 7.82% | 5.12% | 89.24% | **12.96%** | **2.53** | **1.66** |
| 3 | Spanish | 8.14% | 6.52% | 72.95% | **8.76%** | **1.34** | **1.08** |
| 4 | Arab | 5.33% | 4.80% | 64.91% | **4.15%** | **0.86** | **0.78** |
| 5 | Portuguese | 3.91% | 3.21% | 70.99% | **3.91%** | **1.22** | **1.00** |
| 6 | Japanese | 2.77% | 1.75% | 92.63% | **3.47%** | **1.99** | **1.25** |
| 7 | Russian | 3.00% | 2.13% | 82.36% | **3.22%** | **1.51** | **1.07** |
| 8 | Hindi | 3.35% | 4.73% | 41.34% | **2.93%** | **0.62** | **0.88** |
| 9 | French | 1.59% | 1.10% | 84.59% | **2.08%** | **1.89** | **1.31** |
| 10 | German | 1.62% | 1.06% | 89.51% | **1.96%** | **1.85** | **1.21** |

If we only consider first language speakers, French would be in position 9 and quite logically Chinese will show a big advantage over English, despite its very large virtual presence and content productivity. The virtual presence and content productivity for French are very high, despite this ninth place.

Table 14 : Model run with L2 only

|  |  | **Internet users** | **Population** | **Speakers** | **Contents** | **Presence** | **Productivity** |
| --- | --- | --- | --- | --- | --- | --- | --- |
| L2 |  | **L2** | **L2** | **Connected** | **L2** | **Virtual** | **Contents** |
| 1 | English | 32.53% | 31.25% | 55.64% | **37.91%** | **1.21** | **1.17** |
| 2 | Chinese | 8.68% | 6.38% | 72.65% | **10.68%** | **1.67** | **1.23** |
| 3 | French | 6.47% | 5.99% | 57.81% | **6.90%** | **1.15** | **1.07** |
| 4 | Hindi | 6.32% | 8.25% | 40.93% | **5.96%** | **0.72** | **0.94** |
| 5 | Spanish | 3.37% | 2.28% | 78.82% | **5.47%** | **2.39** | **1.62** |
| 6 | Russian | 4.82% | 3.33% | 77.32% | **5.12%** | **1.54** | **1.06** |
| 7 | Malay | 5.37% | 5.21% | 55.08% | **4.52%** | **0.87** | **0.84** |
| 8 | German | 3.10% | 1.87% | 88.72% | **3.61%** | **1.93** | **1.17** |
| 9 | Thai | 1.86% | 1.28% | 77.84% | **1.55%** | **1.21** | **0.83** |
| 10 | Urdu | 1.81% | 5.15% | 18.86% | **1.15%** | **0.22** | **0.63** |
| 11 | Portuguese | 0.68% | 0.81% | 44.81% | **0.89%** | **1.10** | **1.32** |

If we only consider second language speakers, English logically takes a big lead in first place and French takes third place above Spanish.

As a reminder, here are the results for L1+L2.

Table 15: Model results for L1+L2

| **L1** |  | **Internet users** | **Population** | **Speakers** | **Contents** | **Presence** | **Productivity** |
| --- | --- | --- | --- | --- | --- | --- | --- |
| **L2** |  | **L1+L2** | **L1+L2** | **Connected** | **L1+L2** | **Virtual** | **Contents** |
| 1 | Chinese | 18.46% | 14.72% | 71.38% | **21.60%** | **1.47** | **1.17** |
| 2 | English | 14.83% | 13.01% | 64.86% | **19.60%** | **1.51** | **1.32** |
| 3 | Spanish | 6.79% | 5.24% | 73.72% | **7.85%** | **1.50** | **1.16** |
| 4 | Hindi | 4.19% | 5.80% | 41.16% | **3.76%** | **0.65** | **0.90** |
| 5 | Russian | 3.51% | 2.49% | 80.32% | **3.76%** | **1.51** | **1.07** |
| 6 | French | 2.98% | 2.58% | 65.80% | **3.33%** | **1.29** | **1.12** |
| 7 | Portuguese | 2.99% | 2.49% | 68.43% | **3.13%** | **1.26** | **1.05** |
| 8 | Arab | 3.97% | 3.53% | 63.99% | **3.09%** | **0.87** | **0.78** |
| 9 | Japanese | 1.99% | 1.22% | 92.63% | **2.66%** | **2.18** | **1.34** |
| 10 | German | 2.04% | 1.30% | 89.17% | **2.37%** | **1.82** | **1.16** |

A consistency check between the 3 results is made, the third having to flow logically from the first two.

Table 16: Control of L1 and L2 results

|  | WORLD | WORLD | WORLD | ENGLISH | ENGLISH | ENGLISH |  |
| --- | --- | --- | --- | --- | --- | --- | --- |
|  | **POPULATION** | **CONNECTED** | **% CONN.** | **POP.** | **CONN.** | **% CONN.** | Control |
| **L1** | **7,231,699,136** | **4,223,428,027** | **58.40%** | 5.12% | 7.82% | 89.24% | 89.24% |
| **L2** | **3,130,017,620** | **1,673,121,762** | **53.45%** | 31.25% | 32.53% | 55.64% | 55.64% |
| **L1+L2** | **10,361,716,756** | **5,896,549,789** | **56.91%** | 13.01% | 14.83% | 64.86% | 64.86% |
| Control |  |  | 56.91% | 13.01% | 14.83% | 64.86% |  |

In green the checks are carried out: it is a question of calculating the same values ​​directly and thus verifying that the two models L1 and L2 have functioned correctly: the proof is made.

The second series of controls is more complex and one should not expect perfect matches (because modeling is not a linear process with respect to demo-linguistic data).

Table 17: Checking L1 and L2 results (continued)

|  |  | English | Chinese | Spanish | French | Hindi | Portuguese | Russian | German |
| --- | --- | --- | --- | --- | --- | --- | --- | --- | --- |
| Content L1 | | 12.96% | 25.55% | 8.76% | 2.08% | 2.93% | 3.91% | 3.22% | 1.96% |
| L2 content | | 37.91% | 10.68% | 5.47% | 6.90% | 5.96% | 0.89% | 5.12% | 3.61% |
| Contents L1+L2 | | 19.60% | 21.60% | 7.85% | 3.33% | 3.76% | 3.13% | 3.76% | 2.37% |
| Control |  | 20.04% | 21.33% | 7.83% | 3.45% | 3.79% | 3.05% | 3.76% | 2.43% |

The first three lines show the results of the three respective models. The control line in green is calculated by weighting the respective L1 and L2 percentages with respect to the respective connected populations. So, for English, 20.04% is obtained by the following formula: ((12.96 x 4,233,428,027) + (37.91 x 1,673,121,762)) / 5,896,549,789

It is both remarkable and very reassuring, regarding the validity of the model, that the results obtained by the two methods (the L1+L2 model or the prorating of the results of the L1 and L2 models in relation to the respective connected populations) are so close.

Supplementary Material should be uploaded separately on submission. Please include any supplementary data, figures and/or tables.

Supplementary material is not typeset so please ensure that all information is clearly presented, the appropriate caption is included in the file and not in the manuscript, and that the style conforms to the rest of the article.

## ANNEX 10: STRUCTURE OF THE MODEL AND PROCESS

The model is implemented in an Excel file with 17 sheets which are presented below together with the corresponding process.

**ITU**: a copy of ITU source modified according to pre-processing.

**SP**: (stand for SPeakers) the matrix of L1+L2 speakers per country.

In lines, the 329 languages, sorted by 3 digits ISO code (ISO369), starting with line 9 with the sum of the rest of languages not processed.

In columns, the 215 processed countries, sorted by 2 digits ISO code, starting in column I with the sum of the rest of countries not processed.

The 8 first lines and columns are reserved for control information:

Control lines: country code 3 characters, country code 2 characters, country name, total country L1+L2 speakers, % persons connected, number of persons connected, world % connected, total or average (number of languages spoken per country), remaining languages.

Control columns: ISO639, language name^[[4]](#footnote-4)^, total L1+L2 speakers, world % of L1+L2 speakers, world % of L1+L2 connected, number of countries with speakers, number of L1 speakers, ratio L1+L2/L1, remaining countries.

This sheet is **protected from reading** as it contains proprietary information of Ethnologue that cannot be made public.

**SP2**: (Speakers second data) demo-linguistic secondary data computed from **SP**

For the 329 languages in lines, and the rest of languages: world % L1+L2 speakers, number of L1+L2 speakers, world % of connected L1+L2 speakers, number of L1+L2 connected speakers, world % of connected L1+L2 speakers, world % of connected L1 speakers, world % of L1+L2 internauts.

**PL**: (Percentage Language) Matrix parallel to **SP** where PL(i,j) = % of language i internauts from the country j connected, computed from **SP and SP2**. It is a redundant information used to simplify the weighting operation performed in sheet **Wut**.

**MIl**: (Micro-Indicator Language) Holds the list of languages in lines and the value, 0 or 1 of the presence of language in one of the 16 applications used for the *interface* indicator, filled from the sources for languages.

**MIc**: (Micro-Indicator Country) Holds the list of countries in columns and the value, extracted from the external sources for countries, successively for the *index*, *usage* and *traffic* inputs. For version 3 there is 786 lines.

Note that a pre-processing is required for *usage* in order to integrate the non-occidental social networks; this is done in **MIcU** sheet.

Note that a post-processing is required for *traffic* in order to perform the weighting with the optimal number of websites depending of the % of persons connected per country; this is done in **MIcT** and **MIcT1.**

The control columns are the following:

Col. A: indicates the type of indicator from a lookup of the name from **MATRIX**.

Col; B: indicates the name of the indicator

Col. C: depending of the type of data computes the average or the total, or a matrix product with the number of connected persons per country of the inputs in each line

Col. D: indicates the type of data, either a world percentage per country or a quantity per country or a percentage within each country

Col. E: indicates if extrapolation is required and which of the two types of extrapolation if so

Col. F: computes the number of countries with source data

Col. H: hold the URL of the source except for *traffic* where it indicates the number of times the website has been cited, in order to allow a further corresponding weighting in **Wut**.

The control lines are the following:

Line 8 indicates for each country the number of websites which have been measured.

Line 9 indicates the ratio for the number of websites which should have been used in order to respect the proportionality of connected persons (the product line 8 by line 9 for each country represents the number exact of websites required for that country in the hypothesis of the actual total of websites (cell C7) . This will be used as a weighting factor to obtain a fair representation of the traffic measurements in **MIcT1** and **MIcT** prior to the weighting by the number of occurrence of websites done in **Wut** (this have been added in V3C to correct an error in V3 where the weighting as made in parallel with the demo-linguistic weighting).

**MIcU**: (Micro-Indicator Country Usage)The last added sheet included for the new V3 processing of *usage.* Includes a complete copy of the *usage* sources migrated from **MIc**, at the same lines, completed with T-Index and the list of new V3 social networks. For this new list, the traffic measurements per country obtained from SimilarWeb are set, followed by the process of extrapolation (see **EX**). The output is a new and final line called “Social networks weighted” which is obtained by weighting the full list of social networks with the corresponding total of subscribers, balancing finally in a fair manner the occidental social networks with the ones from the rest of the world.

**Ma**: (Mask absence) A sheet parallel at **MIc** holding 1 when a value is absent for the pair (country, input). Used for extrapolation.

**Mp**: (Mask presence) A sheet parallel at **MIc** holding 1 when a value exists for the pair (country, input). Used for extrapolation.

**EX**: (Extrapolation) A sheet parallel at **MIc** where the process of extrapolation is performed. Two different process are used depending of the type of data.

When the data is expressed as a world percentage per country the complement of 100% is split between the countries which have not received data in prorata of their world percentage of connected persons to the Internet. This is typically the case of the *traffic* measurement where the used tools, Alexa and SimilarWeb, do not cover all the countries.

When the data is a rating per country the technics of quartile is used where four quartile values are used depending of where the percentage of connected persons belongs in the interval between : 0%, 15%, 35%, 65%, 85% and 100%.This is typically the case of the *Index* data.

When it appears that either method could not provide meaningful extrapolation the source of data is not included.

In the rare cases where all the countries are informed by the data source obviously no extrapolation is required, as for the NapoleonCat data for percentage of subscribers per social network.

Note that for the V3 process of *usage*, the extrapolation for social networks is not performed in **EX** and has been replicated in **MIcU**.,

Note that the sum of T-Index values for the listed countries is 99.78%, so close to 100% that no extrapolation has been performed.

**MIcT1**: (Micro-Indicator Traffic1) The sheet is parallel at **MIc** and only is filled for the traffic indicators. Each cell (country, website) contains the product of source traffic from **MIc** added with the extrapolated traffic from **EX**, multiplied by the weighting factor for the country from MIP line 10. The sum of percentage is computed and placed in column G for further normalization to 100% in **MIcT**.

**MIcT**: (Micro-Indicator Traffic) The sheet is parallel at **MIcT1** and is used for normalization of the figures to 100% for each website by dividing each cell by the total. The results will be used in **Wut** to compute the final traffic repartition per language.

**Wut**: (Weight usage and traffic) In this sheet the *usage* and *traffic* indicators are processed. The process consists in weighting the values with the percentage of connected speakers per country from **PL**, after applying extrapolation. For the traffic indicator the extrapolation has already been performed in **MIcT** but there is an additional weighting to perform with the figures computed in **MIc** (column H) for the number of occurrences of each website.

**Wi**: (Weight index) In this sheet, the weighting with demo-linguistic figure in order to obtain figures per language is performed for the *index* indicator, starting in column BA, followed, starting in column 10, with the normalization to 100%.

**Pi1**: (Process indicator language) In this sheet, the weighting with demo-linguistic figures is performed, in order to obtain figures per language, for the indicators per country *usage* and *traffic*. For *usage,* an additional weighting is performed with the weight attributed to each component of this indicator (see 2.2.4). For traffic, an additional weighting is performed with the number of occurrences of each website in the sampling (see 2.2.5)

**RES:** (Results) The final results of each indicator per language (usage, traffic, index, interfaces) are computed.

**FINAL**: This sheet presents the final results with all the associated parameters and offers the results sorted for **contents**, **virtual presence**, **content productivity** and **connected speakers**. It also focuses the 20 first content positions and create the cyber-geography of language result (see table 1). A copy without formula of this sheet is made public as the product of the model (see https:/:funredes.org/lc/Results).

**MATRIX**: The list of all the micro-indicators used in the model for each type (*index, interfaces, usages, traffic*).

# Supplementary Figures and Tables

## Supplementary Figures

Figure 1: From sources to products


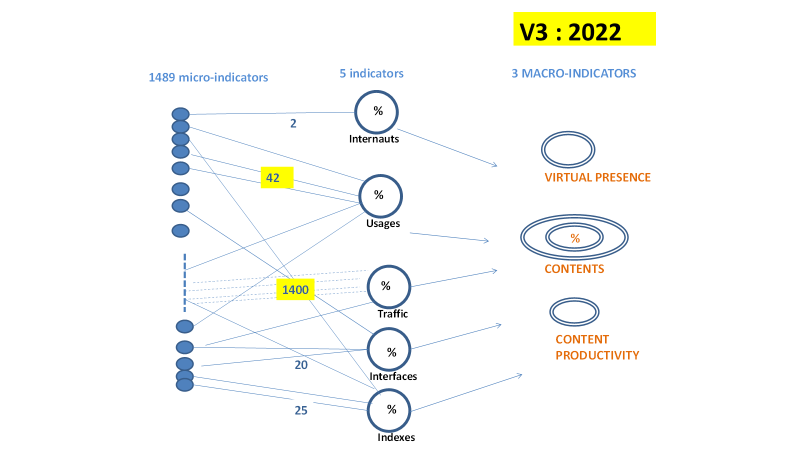


1. Sogou Baike is considered at least as important as Baidu Baké and the same value of number of articles has been assumed. [↑](#footnote-ref-1)
2. https://www.researchgate.net/publication/242100750_Web_Based_Authorship_in_the_Context_of_User_Generated_Content_An_Analysis_of_a_Turkish_Web_Site_Eksi_Sozluk [↑](#footnote-ref-2)
3. <https://www.researchgate.net/publication/>271521393_SOCIAL_MEDIA_IN_A_DICTIONARY_FORMAT_ONLINE_COMMUNITY_OF_eksisozlukcom/figures?lo=1 [↑](#footnote-ref-3)
4. Followed by « macro » if a macro-language. [↑](#footnote-ref-4)
